# A Cosine Similarity Algorithm Method for Fast and Accurate Monitoring of Dynamic Droplet Generation Processes

**DOI:** 10.1038/s41598-018-28270-8

**Published:** 2018-07-02

**Authors:** Xiurui Zhu, Shisheng Su, Mingzhu Fu, Junyuan Liu, Lingxiang Zhu, Wenjun Yang, Gaoshan Jing, Yong Guo

**Affiliations:** 1 Department of Biomedical Engineering, School of Medicine, Collaborative Innovation Center for Diagnosis and Treatment of Infectious Diseases, Tsinghua University, Beijing, China; 2 National Research Institute for Family Planning, Beijing, China; 3 TargetingOne Corporation, Beijing, China; 4 Department of Precision Instrument, School of Mechanical Engineering, State Key Laboratory of Precision Measurement Technology and Instruments, Tsinghua University, Beijing, China

## Abstract

Droplet microfluidics has attracted significant interests in functional microcapsule synthesis, pharmaceuticals, fine chemicals, cosmetics and biomedical research. The low variability of performing chemical reactions inside droplets could benefit from improved homogeneity and reproducibility. Therefore, accurate and convenient methods are needed to monitor dynamic droplet generation processes. Here, a novel Cosine Similarity Algorithm (CSA) method was developed to monitor the droplet generation frequency accurately and rapidly. With a microscopic droplet generation video clip captured with a high-speed camera, droplet generation frequency can be computed accurately by calculating the cosine similarities between the frames in the video clip. Four kinds of dynamic droplet generation processes were investigated including (1) a stable condition in a single microfluidic channel, (2) a stable condition in multiple microfluidic channels, (3) a single microfluidic channel with artificial disturbances, and (4) microgel fabrication with or without artificial disturbances. For a video clip with 5,000 frames and a spatial resolution of 512 × 62 pixels, droplet generation frequency up to 4,707.9 Hz can be calculated in less than 1.70 s with an absolute relative calculation error less than 0.08%. Artificial disturbances in droplet generation processes can be precisely determined using the CSA method. This highly effective CSA method could be a powerful tool for further promoting the research of droplet microfluidics.

## Introduction

Recently, droplet microfluidics is becoming an enabling technology in synthesizing novel materials^1,2^, such as microgels^3–5^, microspheres^6,7^, microcapsules^8^ and quantum dots^9,10^, thus have broad impact in pharmaceutics^11^, fine chemicals^12^, cosmetics^13^ and biomedical research^14,15^. The production of these microscale particles has benefited from the highly uniform droplets generated with the droplet microfluidics^16^, as a low variability of performing chemical reactions inside the droplets could benefit from improved homogeneity and reproducibility^17^. Typically, the uniformity of a droplet generation process is evaluated by the particles’ size distributions after the completion of the process^18,19^. However, various parameters related to the dynamic droplet generation process will result in broader droplet size distributions^20^. Therefore, it is important to develop accurate and convenient methods to monitor dynamic droplet generation processes.

A series of electrical methods were proposed using dielectric properties of emulsions to monitor dynamic generation processes. One of the methods is to embed microelectrodes in microfluidic devices to sense the impedance variations of passing droplets. For instance, Niu *et al*. fabricated 3-D microelectrodes with multi-step photolithography in a microfluidic channel to measure droplet-induced impedance variations^21^. Elbuken *et al*. fabricated non-contact planar microelectrodes on a glass substrate through a lift-off process to measure impedance changes as droplets passing through the channel^22^. Also, radio frequency (RF) devices were applied to monitor droplet generation processes by sensing the changes in resonance frequencies. RF devices were capable of detecting subtle changes in dielectric properties in microfluidic channels^23^. Conchouso *et al*. used RF devices to monitor droplet generation processes in a multi-channel way^16,24^. Although these electrical methods were capable of monitoring both single and multi-channel droplet generation processes, the complexity of chip fabrication and system setup was increased by the requirement of embedded microelectrodes and specialized detection systems.

Another kind of approach to monitoring dynamic droplet generation process is microscopic imaging. These methods use droplet generation video clips acquired by a high-speed camera, which is widely used in a droplet microfluidics experimental system, including droplet generation^25^, observation^26^, and sorting^27^. To keep close tracking of droplets, the high-speed camera is configured at a frame rate over two times of droplet generation or motion frequency. Vladisavljević *et al*. and Park *et al*. monitored the droplet generation processes by manually analyzing the microscopic videos^28,29^. This laborious method is simple and straightforward but with limited accuracy. Beer *et al*. monitored droplet generation processes by calculating the changes in light intensity of an area covered by passing droplets^30^. However, this automatic method is not suitable for monitoring droplet generation processes in multiple channels. Basu proposed a method based on droplet morphometry and velocimetry (DMV) for monitoring droplet generation processes by recognizing droplet interfaces in every frame of the video^31^. Though the DMV method provided various parameters to monitor droplet generation processes, recognizing droplet interfaces in every video frame may require a sizeable computational resource, and the monitoring may not keep pace with droplet generation processes. In general, though microscopic imaging methods do not require specialized equipment or elaborate chip fabrication, they are not capable of monitoring dynamic droplet generation processes in an accurate and online manner.

Many progress has been made to monitor the dynamic droplet generation processes. However, accurate, convenient and online methods have rarely been described. In this paper, we proposed a novel cosine similarity algorithm (CSA) method for automatic monitoring of dynamic droplet generation processes. Through acquiring microscopic video clips of droplet generation processes, the CSA method was able to calculate droplet generation frequency distributions from the periodic changes of cosine similarities between video frames. Both differences among multiple channels and changes along the timeline could be closely monitored by the CSA method in a fast, convenient and accurate manner with differences among frequency peaks and changes in frequency distributions. To the best of our knowledge, this is the first time that cosine similarity has been applied to monitor dynamic droplet generation processes in droplet microfluidics, and this highly effective method could be a powerful tool for further promoting the research and development of droplet microfluidics.

## Results and Discussion

### Schematic Diagram of the Cosine Similarity Algorithm (CSA)

**Figure 1:**
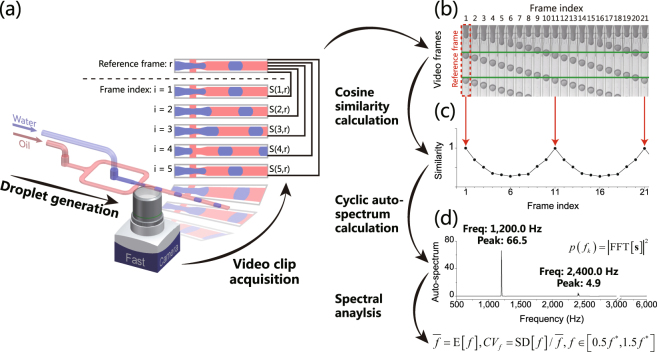
Schematic diagram of the cosine similarity algorithm (CSA) method for the calculation of droplet generation frequency’s mean value and coefficient of variation (CV). (**a**) Droplets are generated with a microfluidic chip, and a microscopic video clip is captured by a high-speed camera. (**b**) A reference frame is designated as shown in the box with red dashed border. (**c**) Similarity vector is calculated consisting of the cosine similarities between each frame (including the reference frame) and the reference frame. The oscillation of the similarity vector is correlated with the generation of droplets. (**d**) The cyclic auto-spectrum reveals the oscillation frequency of the vector, and the fundamental frequency peak at frequency f* = 1,200.0Hz is converted to droplet generation frequency’s mean value $\bar{f}$ and CV *CV*_*f*_ (see supplementary methods in the supplementary information for further details about the equations).

Typically, cosine similarity is applied to characterize the similarity between two images, which is widely used in various image-processing applications, such as face recognition^32^ and template matching^33^. As shown in Fig. 1, the CSA method consists of four steps. First, a microscopic droplet generation video clip is captured at a constant frame rate with a high-speed camera. Second, frames of the same spatial resolution are extracted from the acquired video clip. Among these frames, one of them is designated as the reference frame. The cosine similarity is calculated between the reference frame and each frame in the video clip, including the reference frame with itself. Third, a periodic similarity vector is constructed to combine the calculated similarities with the frame indices in ascending order. Since a stable droplet generation process is highly periodic, the similarity vector oscillates with the periodic reappearance of the reference frame. Finally, cyclic auto-spectrum is calculated with fast Fourier transform (FFT) to obtain the frequency distribution of the similarity vector, through which the mean value and coefficient of variation (CV) of the droplet generation frequency can be calculated from the further spectral analysis.

### Principle of CSA

Mathematically, cosine similarity between two non-zero vectors **a** and **b** is defined as the cosine of the included angle 〈***a***, ***b***〉^34^, which is given by equation (1):

1 $$S({\bf{a}},\,{\bf{b}})\triangleq \,\cos \langle {\bf{a}},\,{\bf{b}}\rangle =\frac{{{\bf{a}}}^{{\rm{T}}}{\bf{b}}}{{\rm{\Vert }}{\bf{a}}{\rm{\Vert }}{\rm{\Vert }}{\bf{b}}{\rm{\Vert }}}.$$

The range of cosine similarity is determined by the cosine function: *S*(**a**, **b**) ∈ [−1, 1]. The larger the |*S*(**a**, **b**)|, the higher the non-zero vectors **a** and **b** are linearly correlated (“similar”). For two grayscale frames with the same spatial resolution, cosine similarity can be used to characterize the similarity between the frames^35^. The grayscales of the pixels in each frame can be arranged into a grayscale vector in a specific order. For instance, for a grayscale frame (spatial resolution: *m* × *n* pixels) with index *i* in which the grayscale of pixels are defined as *H*(*p*, *q*, *i*), *p* = 1, 2, …, *n*, *q* = 1, 2, …, *m*, the grayscale vector **h**(*i*) can be defined as shown in equation (2):

2 $${\bf{h}}(i)\triangleq [\begin{array}{c}H(1,1,i)\\ H(1,2,i)\\ \vdots \\ H(1,m,i)\\ H(2,1,i)\\ H(2,2,i)\\ \vdots \\ H(2,m,i)\\ \vdots \\ H(p,q,i)\\ \vdots \\ H(n,1,i)\\ H(n,2,i)\\ \vdots \\ H(n,m,i)\end{array}].$$

**Figure 2:**
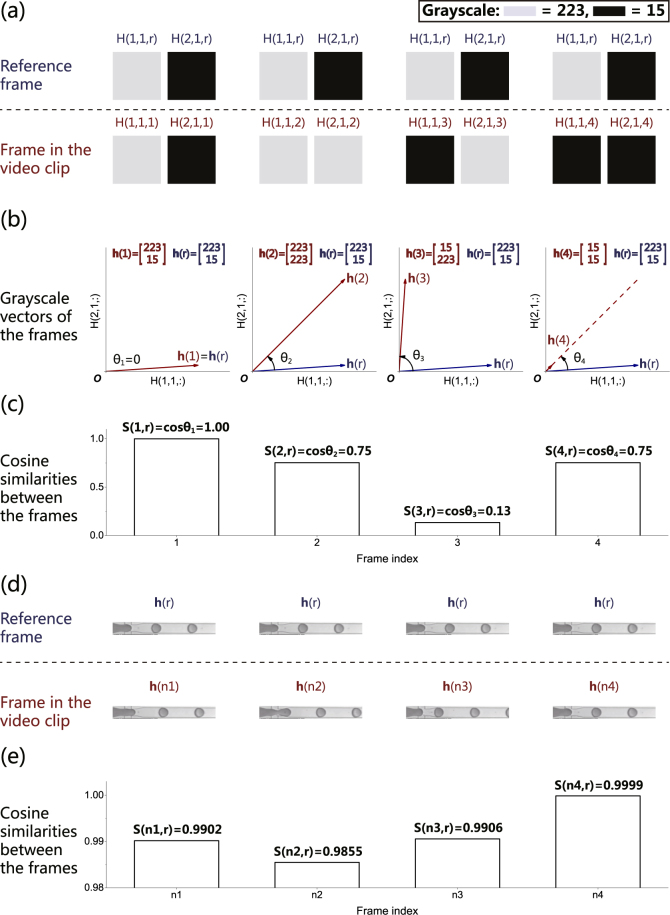
The concept of cosine similarity and its calculation. (**a**) A reference frame and four frames from a video clip. The frames (with indices 1, 2, 3 and 4) have a spatial resolution of 2 × 1 pixels. Frame 1 is designated as the reference frame (with index r). (**b**) The grayscale vectors **h**(1), **h**(2), **h**(3), **h**(4), and their corresponding included angles *θ*_1_, *θ*_2_, *θ*_3_, *θ*_4_ with the grayscale vector **h**(*r*) are illustrated. (**c**) The cosine similarities are calculated as the cosine of the included angles. (**d**) Four frames (with indices *n*1, *n*2, *n*3 and *n*4 in ascending order) and a reference frame (with index *r* = 1; *n*1) from a droplet generation video clip. (**e**) The cosine similarities are calculated between each frame from the video clip and the reference frame, indicating that frame *n*4 was identical to the reference frame.

Figure 2a illustrates a simplified example of four grayscale frames (spatial resolution: 2 × 1 pixels) with indices *i* = 1, 2, 3, 4. A grayscale vector of each frame can be defined according to equation (2). If frame 1 is designated as the reference frame (with index defined as *r*), then the cosine similarities between each of the four frames and the reference frame can be calculated as the cosines of the included angles illustrated in Fig. 2b. As shown in Fig. 2c, the highest similarity of 1.00 is between the reference frame and itself. The similarity between the reference frame and each frame is positively correlated with the calculated cosine similarity: the more similar the frames, the higher the cosine similarity.

Similarly illustrated in Fig. 2d, four grayscale frames (resolution: 512 × 62 pixels) with indices *n*1, *n*2, *n*3 and *n*4 are selected along the timeline of a droplet generation video clip. Frame 1 of the video clip is designated as the reference frame (with index *r* = 1), and the similarities between each of the four frames and the reference frame are calculated (Fig. 2e). Notably, the similarity between the reference frame and frame *n*4 (0.9999) is higher than those between the reference frame and the other frames (0.9902, 0.9855, 0.9906, respectively for frame *n*1, *n*2, and *n*3), indicating the reappearance of the reference frame along the droplet generation process.

Our hypothesis is that a stable droplet generation process is a highly periodic process. Once a reference frame is designated and the cosine similarities are calculated between the reference frame and each frame in the video clip, a cosine similarity vector is constructed with these calculated similarities. The value of the cosine similarity in the vector oscillates periodically. Thus, the oscillating frequency of the similarity vector, which is correlated with droplet generation frequency, can be derived from further spectral analysis. The detailed mathematic derivation can be obtained at supplementary methods in the supplementary information.

### Four kinds of dynamic droplet generation processes were monitored using CSA

With the novel CSA method, four kinds of dynamic droplet generation processes were monitored. The CSA method was applied (1) to calculate droplet generation frequency in a stable condition in a single microfluidic channel, (2) to calculate non-overlapping droplet generation frequencies in a stable condition in multiple microfluidic channels simultaneously, (3) to monitor the droplet generation process with artificial disturbances in a single microfluidic channel, and (4) to monitor pre-microgel droplet generation processes online.

### Case 1. Droplet generation process in a stable condition in a single microfluidic channel

**Figure 3:**
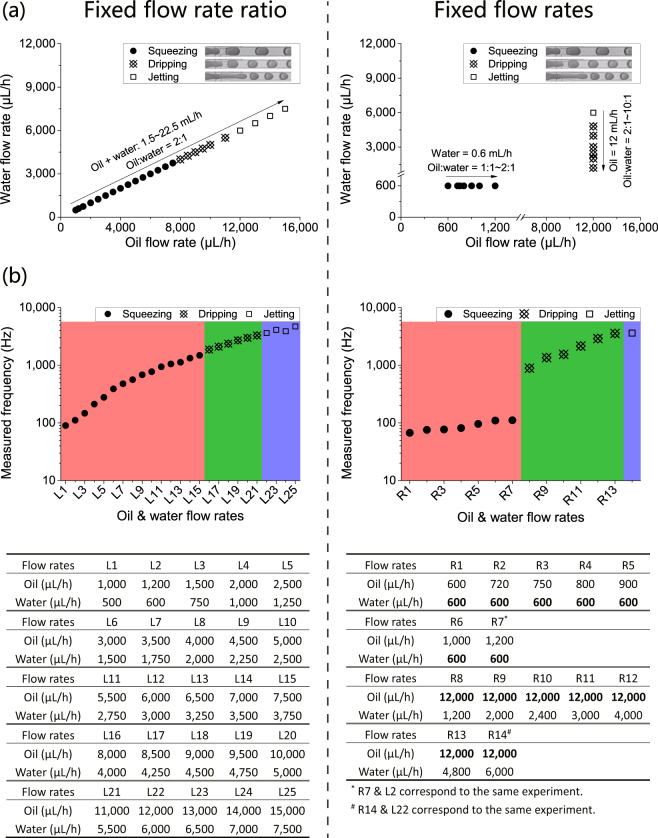
Two configurations of flow rates: fixed oil/water flow rate ratio and fixed oil and water flow rates, were investigated for droplet generation processes in a stable condition in single microfluidic channels. The droplet was generated (**a**) in three different modes: squeezing, dripping and jetting, and (**b**) at different frequencies ranging from 67.3 Hz to 4,707.9 Hz. The labels in the x-axes correspond to different combinations of oil and water flow rates shown at the bottom of the figure.

Using the CSA method, accurate calculation of droplet generation frequency’s mean value and CV can be obtained for a droplet generation process in a stable condition in a single microfluidic channel (Table S1). Typically, there are three different droplet generation modes in a stable droplet generation process, including squeezing, dripping and jetting^36^. As shown in Fig. 3a, two flow rate configurations (fixed flow rate ratio and fixed flow rates) were used to evaluate the performance of the CSA method in these droplet generation modes.

As shown in Fig. 3b, droplet generation frequency increased from 90.1 Hz to 4,707.9 Hz as the total oil and water flow rate increased from 1.5 mL/h to 22.5 mL/h, with an oil/water flow rate ratio fixed at 2:1. The droplet generation mode switched from squeezing to dripping, and then to jetting at critical droplet generation frequencies of 1,481.6–1,874.8 Hz and 3,287.3–3,614.2 Hz, respectively. When the water flow rate was fixed at 0.6 mL/h, droplet generation frequency increased from 67.3 Hz to 110.2 Hz in the squeezing mode as the oil/water flow rate ratio increased from 1:1 to 2:1. However, when the oil flow rate was fixed at 12 mL/h, droplet generation frequency increased from 892.0 Hz to 3,563.1 Hz in the dripping and jetting modes as the oil/water flow rate ratio decreased from 10:1 to 2:1.

**Figure 4:**
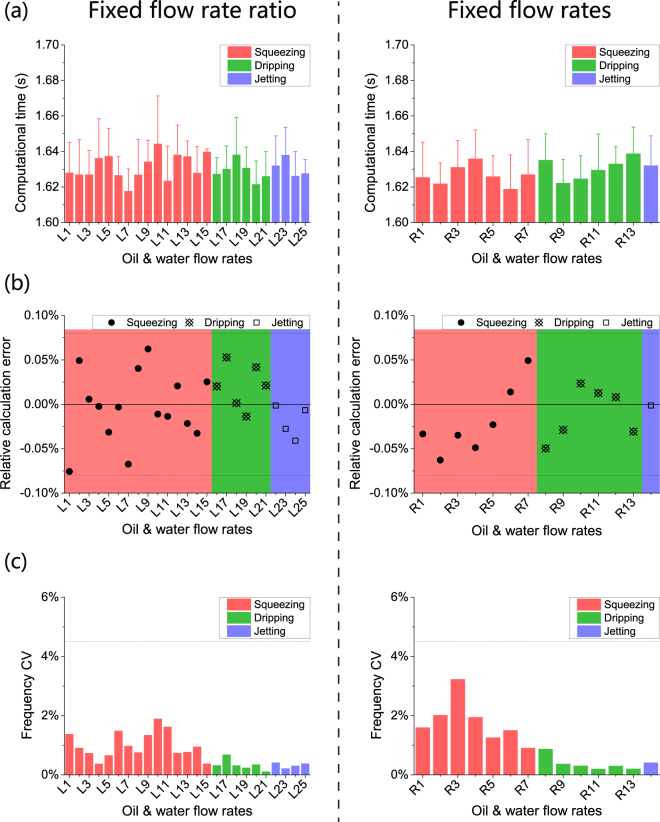
With the CSA method, fast and accurate calculation of droplet generation frequency’s mean value and CV can be obtained for droplet generation processes in a stable condition in single microfluidic channels. (**a**) The average computation time of CSA was 1.63 s. (**b**) The absolute relative calculation error was less than 0.08%. (**c**) Droplet generation frequency’s CV indicated the size uniformity of droplets in the three different modes, and droplets generated in dripping mode had the highest size uniformity with an average CV of 0.35%. The labels in the x-axes correspond to different combinations of oil and water flow rates were shown at the bottom of Fig. 3.

With the CSA method, the calculation is fast and accurate. As shown in Fig. 4a, the average computational time was 1.63 s for a video clip with 5,000 frames at a resolution of 512 × 62 pixels, with a computation speed of about 3,000 frames per second (fps). As shown in Fig. 4b, the absolute relative calculation error of droplet generation frequency was less than 0.08% compared with that obtained by the manual counting-timing method. Figure 4c showed that the average CV of droplet generation frequency of the dripping mode was 0.35%, which was smaller than that of the squeezing and jetting modes, although the differences were not statistically significant. It suggested that droplets generated in the dripping mode were more uniform than those generated in the other two modes. This conclusion is in accordance with the average droplet diameter’s CV in the published literature^37^.

### Case 2. Droplet generation process in a stable condition in multiple microfluidic channels

The CSA method can also be used to evaluate droplet generation frequencies in a stable condition in multiple microfluidic channels (Table S2), a widely used droplet microfluidic technique for specific applications^38,39^.

**Figure 5:**
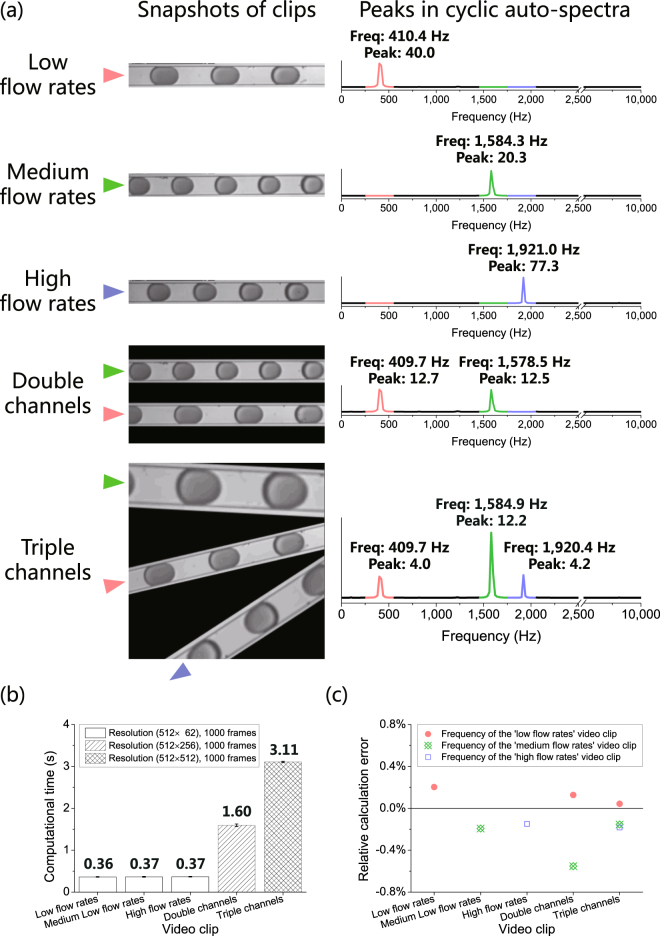
With the CSA method, fast and accurate calculation of droplet generation frequency’s mean value and CV can be obtained for droplet generation processes in a stable condition in multiple microfluidic channels. For droplet generation processes in a stable condition in multiple channels, (**a**) three droplet generation processes in a stable condition in single microfluidic channels were analyzed with CSA as non-overlapping droplet generation frequencies and two droplet generation processes in a stable condition in multiple microfluidic channels were reconstructed. The CSA method calculated all the non-overlapping droplet generation frequencies in the two reconstructed video clips in single runs. The positions of frequency peaks in the two reconstructed video clips were consistent with those in the three corresponding droplet generation processes in single channels. (**b**) The average computation time of CSA increased from 0.36 s to 3.11 s with the increase in spatial resolution of the video clips. (**c**) The absolute relative calculation error of the droplet generation processes in a stable state in single channels was less than 0.21%, and that of the droplet generation processes in a stable state in multiple channels was less than 0.56%.

As shown in Fig. 5a, three droplet generation processes with different flow rates (low, medium and high) in a stable condition in single microfluidic channels were analyzed with the CSA method, and the calculated droplet generation frequencies were 410.4 Hz, 1,584.3 Hz, and 1,921.0 Hz, respectively, with no overlapping in the cyclic auto spectrum. Next, to mimic the droplet generation process in multiple microfluidic channels, two video clips were reconstructed: a double-channel droplet generation process and a triple-channel droplet generation process. For a double-channel droplet generation process, two single-channel droplet generation video clips in a stable condition were combined to form a video clip of two parallel microfluidic channels with the same width. The CSA method generated an auto-spectrum with two distinctive peaks at 409.7 Hz and 1,578.5 Hz in a single run. For a triple-channel droplet generation process, three single-channel droplet generation video clips in a stable condition were zoomed, rotated and combined to form a video clip of three microfluidic channels with different widths and directions. The CSA method generated an auto-spectrum with three distinctive peaks at 409.7 Hz, 1,584.9 Hz, and 1,920.4 Hz in a single run. Therefore, the cyclic auto spectra of both configurations yielded droplet generation frequencies consistent with the corresponding single-channel droplet generation processes that made up the video clips.

The CSA method can obtain multiple droplet generation frequencies in a single run. The reason is that partial similarity between two frames contributes to the cosine similarity between them, so each of the microfluidic channels makes corresponding oscillating contributions to the similarity vector at their own, non-overlapping frequencies. These contributions will be converted into different frequency peaks in the cyclic auto-spectrum.

As shown in Fig. 5b, the average computational time of CSA was approximately proportional to the number of frames and spatial resolution of the video clip. For video clips with a spatial resolution of 512 × 62 pixels and 1,000 frames, which is 20% of the number of frames in case 1, the average computational time was 0.37 s, which was 23% of the computational time in case 1. The two reconstructed video clips increased the spatial resolution to about four and eight times of the original spatial resolution, resulting in an increase in computational time from 0.37 s to 1.60 s and 3.11 s respectively, which were 4.3 and 8.5 times of the computational time for a video clip with a spatial resolution of 512 × 62 pixels.

As shown in Fig. 5c, the absolute relative calculation error for the single microfluidic channels was less than 0.21%, and the error for the multiple microfluidic channels was less than 0.56%. The calculation results of the two droplet generation processes in multiple microfluidic channels were referred to the frequencies of their corresponding single-channel processes for the calculation of relative calculation error. Notably, the largest absolute relative error, in this case, was larger than that in case 1 (0.08%). This was due to the reduction of the number of frames in a video from 5,000 in case 1 to 1,000 in this case, which resulted in a reduction in the spectral resolution to 20% compared with that in case 1. Due to the reduction in spectral resolution, the absolute relative calculation error was accordingly expected to increase up to five times of the calculated error in case 1^40^, which was 0.40% in this case. As shown in Fig. 5c, the absolute relative calculation error in this case for single-channel processes was less than 0.21%, which was less than the maximum error of 0.40%. Furthermore, since reconstruction of video clips did not result in a linear addition of similarity vectors, crosstalks between frequency components (both fundamental and harmonic frequencies) might result in an increase in the absolute relative calculation error, which could lead to an increase in the error from 0.21% to 0.56% for droplet generation processes in the multiple microfluidic channels.

### Case 3. Droplet generation process with artificial disturbances in a single microfluidic channel

Using the CSA method, droplet generation process with artificial disturbances in a single microfluidic channel have been closely monitored, with the periods affected by the disturbances precisely determined (Table S3). Artificial disturbances can be introduced into droplet generation process by changing the oil or water flow rates, which resulted in droplet size variation, generation frequency and spacing^41^.

**Figure 6:**
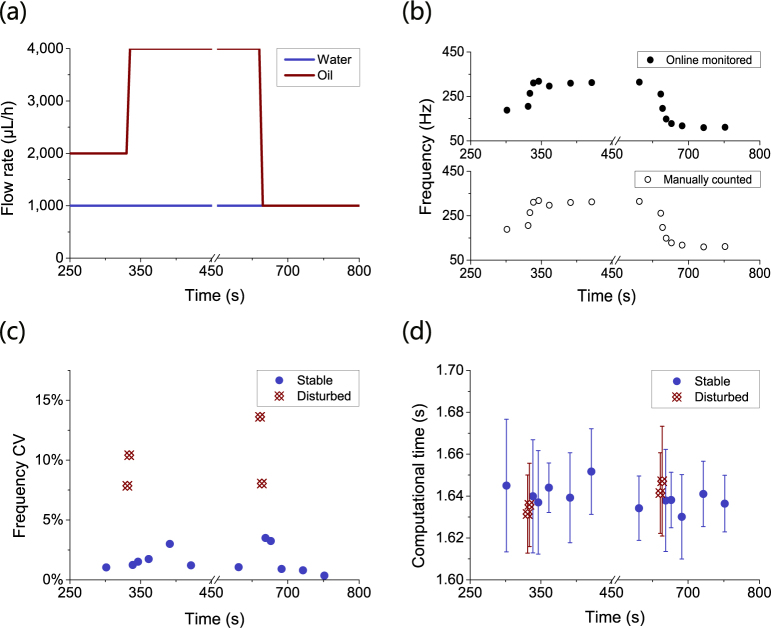
The time periods affected by artificial disturbances can be detected and located with the CSA method in a droplet generation process. For a droplet generation process with artificial disturbances in a single channel, (**a**) the artificial disturbances were introduced by toggling oil flow rate from 2,000 μL/h to 4,000 μL/h and finally to 1,000 μL/h during the course of the droplet generation process. (**b**) The droplet generation frequency’s mean value was closely monitored online with CSA. (**c**) Droplet generation frequency’s CV increased from 1.63% to 9.98% on average when the disturbances occurred. (**d**) The average computation time was 1.64 s, which was not affected by the disturbances.

As shown in Fig. 6a, a droplet generation process with changing oil flow rates was used to investigate droplet generation process in a single channel with artificial disturbances. The whole process included three stable stages separated by two artificial disturbances, with each stable stage of 5.50 min and each disturbance stage of 5.00 s. As shown in Fig. 6b, changes in droplet generation frequency could be monitored closely at an interval as short as 2.50 s (the duration of the process in a video clip) with the CSA method. Over the course of the droplet generation process, droplet generation frequency’s mean value calculated with the CSA method synchronized with the measured mean droplet generation frequency without delay. As shown in Fig. 6c, an increase in droplet generation frequency’s CV was observed twice, from an average of 1.63% to 9.99%, which was consistent with the two periods affected by the disturbances. As shown in Fig. 6d, the average computational time was 1.64 s, which was not affected by the disturbances, because the CSA method did not differentiate any graphical feature in the video clip. As long as the disturbances did not affect the computational scale (for example, number of frames and spatial resolution), it did not affect the CSA computational time.

Thus, a droplet generation process with artificial disturbances can be closely monitored with the CSA method. The calculated mean frequency changes promptly after a change in the actual droplet generation frequency, and the CV indicated whether the droplet generation process is disturbed.

### Case 4. Pre-microgel droplet generation processes with or without artificial disturbances

Microgel is widely used in biology^42–44^ and material science^45,46^ because of its good biocompatibility and deformability^47^. Highly monodispersed pre-microgel droplets can be generated in a high-throughput manner^48^, and they serve as a prerequisite for the fabrication of highly monodispersed microgels^49^. Droplet generation frequency is a very effective parameter to closely monitor dynamic microgel generation processes.

**Figure 7:**
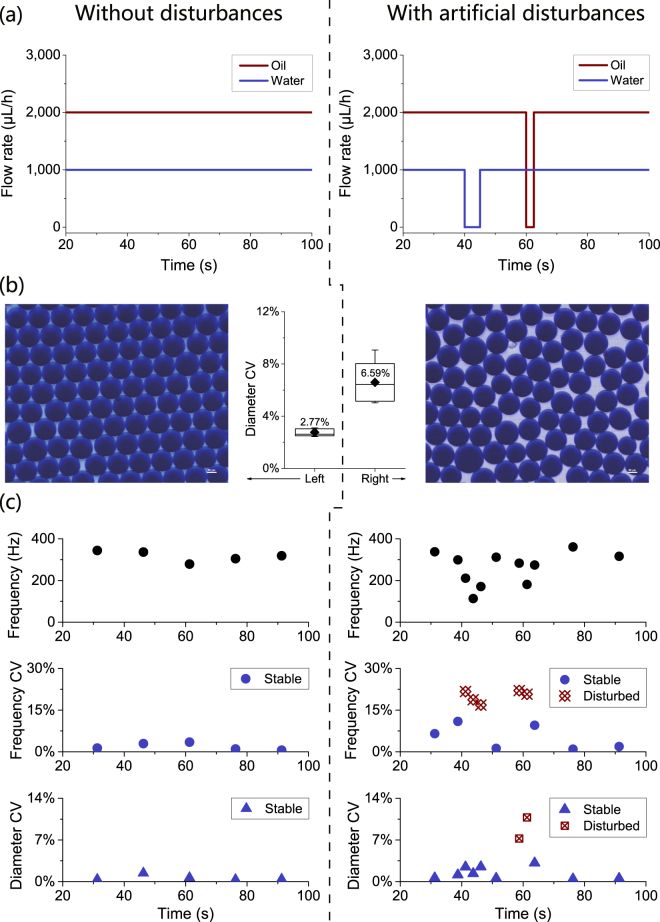
The size uniformity of pre-microgel droplets can be characterized and monitored with the CSA method in pre-microgel droplet generation processes with or without disturbances. For a pre-microgel droplet generation process, (**a**) two configurations of oil and gel solution flow rates were investigated: one without disturbances and the other with intentional interruptions of oil and water phase injection as artificial disturbances. (**b**) Although droplet diameter’s mean value was not affected by the disturbances to a large extent, droplet diameter’s CV (calculated from microscopic images) increased from 2.77% to 6.59% on average due to the disturbances. The scale bars in the microscopic images are 50 μm. (**c**) Droplet generation frequency was closely monitored with CSA in both configurations. The size uniformity of the pre-microgel droplets was characterized by two different parameters: droplet generation frequency’s CV and droplet diameter’s CV. In the configuration without disturbances, both parameters indicated no disturbances. In the configuration with artificial disturbances, however, droplet generation frequency’s CV indicated that artificial disturbances had happened twice during the process, while droplet diameter’s CV indicated only once.

As shown in Fig. 7a, two microgel generation processes were investigated with the CSA method: one without artificial disturbances, and the other with intentional interruptions of oil and water injection (once each) as artificial disturbances. The two processes were monitored based on both droplet generation frequency and droplet size (Table S4). Droplet generation frequency and its CV were monitored with the CSA method, and the droplet size and its CV were calculated with a custom Matlab program. Although the disturbances did not change the droplet diameter’s mean value drastically (116.1 μm and 117.2 μm for the two processes respectively, as shown in Table S5), they significantly increased the droplet diameter’s overall CV, from 2.77% to 6.59%, resulting in a wider size distribution and lower monodispersity.

As shown in Fig. 7b,c, droplet generation frequency’s CV varied synchronously with both periods affected by the interruptions of oil and water injection, while droplet diameter’s CV varied synchronously with the period affected by the interruption of oil injection only. Similar to case 3, the droplet generation frequency’s CV revealed all the disturbances. In the process without disturbance, the droplet generation frequency’s CV remained stable, and so did the droplet diameter’s CV. On the contrary, in the processes with artificial disturbances, the droplet generation frequency’s CV varied synchronously with both periods affected by the disturbances, but the droplet diameter’s CV did so with only one.

The reason why the changes in droplet diameter’s CV failed to reveal both disturbances is that the droplet diameter is mainly related to the oil flow rate^50^. During the interruption of water phase injection, droplet diameter did not undergo changes large enough for an obvious increase in droplet diameter’s CV. In contrast, droplet generation frequency is related to both oil and water flow rates^50^, and the interruption of oil or water injection managed to change droplet generation frequency to an extent large enough for an obvious increase in droplet generation frequency’s CV. In this way, droplet generation frequency’s CV serves as a better parameter than droplet diameter’s CV to indicate interruptions of oil or water injection, which may be encountered in various cases during a pre-microgel droplet generation process, including power failure, equipment glitches, depletion of oil or water phase and clogging of microfluidic channels.

The current CSA method is an online approach, which updates computational results of droplet generation frequency at a designated interval (for example, every 2.50 s). It should be a great improvement if the CSA method could monitor droplet generation frequency in real time. In order to achieve real-time droplet generation frequency monitoring, a few modifications are needed for the CSA method. First, two data buffers with specific sizes are set up to store captured video frames and the cosine similarities between the respective video frames and the reference frame. The calculated cosine similarities form a similarity vector. Both data buffers are “first in, first out” (FIFO) buffers. For instance, a FIFO data buffer is configured to store 5,000 video frames maximally. Before the data buffer is filled up with 5,000 video frames, there is a build-up phase in which newly captured video frames are stored in the data buffer continuously. Once the data buffer is full of 5,000 video frames, it switches to a real-time phase with the “first in, first out” principle. For instance, a 5,001^st^ video frame is stored into the data buffer synchronized with the deletion of the 1^st^ video frame. The same principle is also applied for the data buffer storing respective calculated cosine similarity between the newly acquired frame and the reference frame. During the build-up phase, the number of data buffer with calculated cosine similarities increases, but the calculated cyclic auto-spectrum and droplet generation frequency have limited accuracy due to insufficient data. While during the real-time phase, the droplet generation frequency calculation is updated with each newly captured video frame. Without the two FIFO data buffers, the computational time of similarity vector is *O*(*N*_Frames_*R*) in terms of time complexity (*N*_Frames_: the number of video frames, *R*: the spatial resolution of each frame), and the computational time of cyclic auto-spectrum of the similarity vector is *O*(*N*_Frames_log_2_*N*_Frames_) in terms of time complexity^51^. Therefore, the computational time for droplet generation frequency is mainly determined by the calculation of the similarity vector, not the calculation of cyclic auto-spectrum. With the aid of the two FIFO data buffers, the computational time for droplet generation frequency is greatly reduced to 1/5,000 of that without using the two data buffers, since only one cosine similarity needs to be calculated per update when using the data buffers, while 5,000 cosine similarities need to be calculated per update otherwise. We did a repetition of ten computations during the real-time phase, and the computational time was 2.92 × 10^−4^ ± 2.31 × 10^−5^ s, which saved about 99.98% the average computational time (1.76 s) without these two data buffers. For a time lapse of 5 × 10^−4^ between two frames at the frame rate of 2,000 fps, the computational time 2.92 × 10^−4^ ± 2.31 × 10^−5^ s is short enough to achieve real-time droplet generation frequency monitoring. We believe the modified CSA method can be applied to droplet frequency calculation at higher frame rates with the increased capability of the high-speed camera and computational resource. The current report can provide impetus toward real-time calculation of droplet generation frequency for a closer monitoring of dynamic droplet generation processes.

## Conclusions

In summary, a novel Cosine Similarity Algorithm (CSA) method was developed to monitor dynamic droplet generation processes. With a microscopic droplet generation video clip at a constant frame rate captured by a high-speed camera, four kinds of dynamic droplet generation processes were monitored. Single-channel droplet generation processes were monitored in about 1.63 s on average with an absolute relative calculation error less than 0.08%. Three different stages of droplet generation, squeezing, jetting and dripping, could be differentiated using droplet generation frequency’s CV calculated by the CSA method. Multi-channel droplet generation processes were monitored in 0.36–3.11 s with an absolute relative calculation error less than 0.56%. Meanwhile, the periods affected by the disturbances were accurately determined by droplet generation frequency’s CV. For pre-microgel droplet generation processes with or without artificial disturbances, the results of dynamic monitoring conformed with droplet size uniformities along the timeline. The experimental results demonstrated the effectiveness of the CSA method for monitoring dynamic droplet generation processes in an accurate, convenient and online manner. The CSA method could be a powerful tool for further promoting the research and development of the droplet microfluidics.

## Materials and Methods

### Materials and reagents

PDMS (polydimethylsiloxane) base and its curing reagent (Slygard 184) were purchased from Dow Corning (Midland, MI). Aquapel was purchased from PPG Industries (Pittsburgh, PA). Novec 7500 was purchased from 3 M Inc. (St. Paul, MN). FluoroSurfactant was purchased from RAN Biotechnologies (Beverly, MA). Brilliant blue was purchased from Shanghai Dyestuff Research Institute Co. Ltd. (Shanghai, China). Acrylamide, bis-acrylamide, APS (ammonium persulfate) and TEMED (tetramethylethylenediamine) were purchased from Aladdin Reagent Inc. (Shanghai, China).

### Fabrication of droplet generation chip

The droplet generation chip was designed as a flow-focusing droplet generator whose nozzle was 70 μm wide and 70 μm high. The droplet generation chip was fabricated with standard soft lithography as described earlier^52^. Briefly, PDMS base and its curing agent were thoroughly mixed and degassed in a vacuum oven (BZF-30, Shanghai Boxun Industry & Commerce Co. Ltd., China). Next, the mixture was cast onto an SU-8 mold fabricated with conventional photolithography (CapitalBio Corporation, China) and cured at 80 °C for 55 min in an oven (DFZ, Beijing Zhongxingweiye Instrument Co. Ltd., China). Then, the PDMS slab was peeled off the SU-8 mold, punched for inlets and outlets, and bonded to a glass substrate with oxygen plasma treatment (Femto, Diener Electronic, Germany).

### Droplet generating and monitoring platform

**Figure 8:**
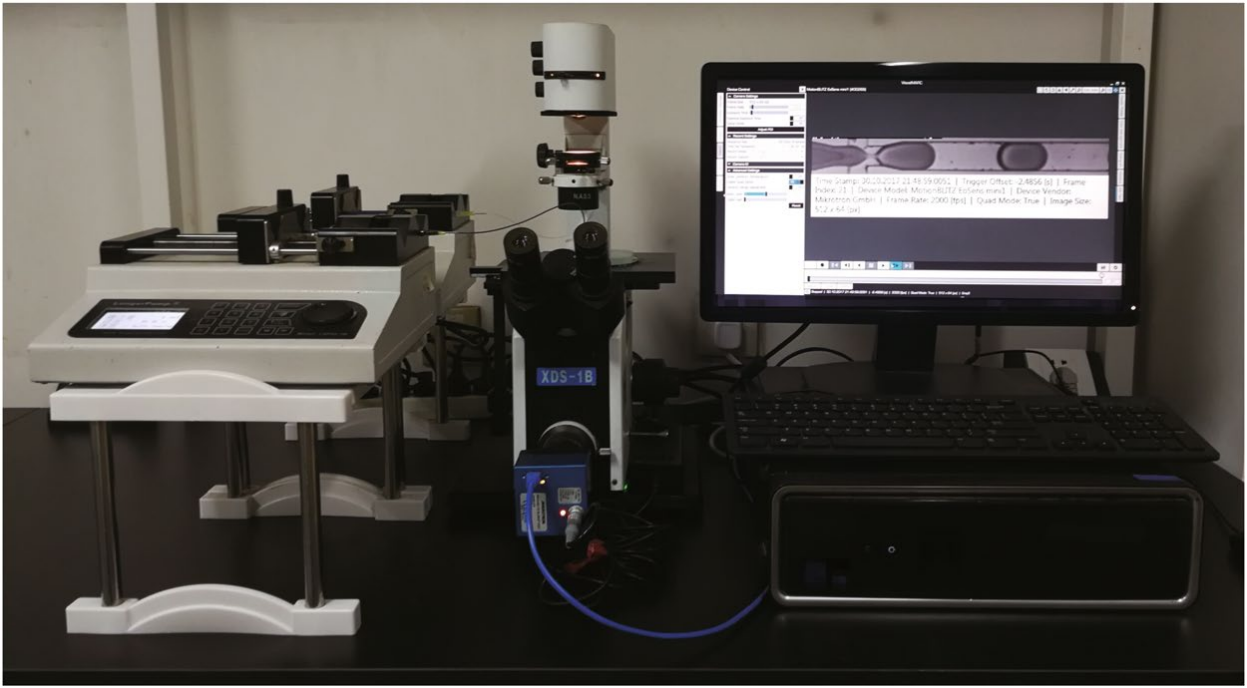
Droplet generating and monitoring platform. Droplets were generated with a conventional droplet generation platform consisting of two syringe pumps, a microscope, a drop-maker microfluidic chip, a high-speed camera, a personal computer, syringes, and tubings.

A conventional droplet generating and monitoring platform was established as described earlier (Fig. 8)^53^. The droplet generation chip was placed on the stage of an inverted microscope (XDS-1B, Chongqing Optical Instrument Corporation, China). Two syringe pumps (LSP02-1B, Longer Precision Pump Co. Ltd., China) were used to inject the oil phase and the water phase into droplet generation chip through the inlets. Video clips of the droplet generation processes were captured with a high-speed camera (MotionBlitz EoSens 1, Mikrotron, Germany).

### Droplet generation experiments

In the pre-microgel droplet generation experiment, crosslinker premix was used as the oil phase, 2% (w/w) FluoroSurfactant and 0.4% (v/v) TEMED dissolved in Novec 7500. Meanwhile, acrylamide premix was used as the water phase, consisting of 6.2% (w/v) acrylamide, 0.18% (w/v) bis-acrylamide, 0.3% (w/v) APS and 7% (w/v) brilliant blue dissolved in double-distilled (DI) water. In the other droplet generation experiments, Novec 7500 was used as the oil phase, and 7% (w/v) brilliant blue dissolved in DI water was used as the water phase to visualize the droplets. In all experiments, the droplets were generated with the droplet generation chip by injecting the oil and water phases at designated flow rates (Table S1–S4) into the designated inlets. The spatial resolution of the video clip was set at 512 × 62 pixels, and the frame rate of the high-speed camera was configured to over five times of droplet generation frequencies. The microscopic video clips were acquired with the high-speed camera at designated time points from the start of droplet generation (Table S1–S4). 5,000 frames in each acquired video clip were selected and saved in H264 coding format. The video clips acquired with the high-speed camera were converted to AVI files with the VideoWriter module in Matlab (Mathworks Inc., Natick, MA).

### Reconstruction of video clips

Video clips for droplet generation process in multiple channels were reconstructed from the video clips from droplet generation process in a single channel with Premiere Pro CS6 (Adobe Inc., Mountain View, CA). The first 1,000 frames of each video clip were selected. For a video clip for droplet generation process in two channels, two single-channel video clips were reconstructed to form a video clip with two parallel microfluidic channels of the same width, resulting in a two-channel droplet generation video clip with a spatial resolution of 512 × 256 pixels. For a video clip for droplet generation process in three channels, three single-channel video clips were reconstructed by zooming (110%, 200%, and 150%, respectively), rotating (−13°, 3°, and 147°, respectively) to form a video clip with three microfluidic channels of different widths and directions, resulting in a three-channel droplet generation video clip with a spatial resolution of 512 × 512 pixels. For the other droplet generation processes, no reconstruction was required.

### Processing of monitor results

The mean frequencies and their CVs of each droplet generation process were automatically calculated with a customized Matlab program based on the CSA method. Meanwhile, droplet diameter CVs of the pre-microgel droplets were automatically analyzed with another customized Matlab program based on droplet interface recognition in microscopic images acquired after the droplet generation processes.

### Data availability Statement

The datasets generated during and/or analyzed during the current study are available from the corresponding author on reasonable request.

## Electronic supplementary material

Electronic Supplementary Information
