# Supplementary material for: A Cosine Similarity Algorithm Method for Fast and Accurate Monitoring of Dynamic Droplet Generation Processes
Source: Sci Rep. 2018 Jul 2;8:9967. doi: 10.1038/s41598-018-28270-8 (PMC6028520; doi:10.1038/s41598-018-28270-8)
Supplement: Supplementary file 1 — Electronic Supplementary Information [file 41598_2018_28270_MOESM1_ESM.docx]

Supplementary Information

A Cosine Similarity Algorithm Method for Fast and Accurate Monitoring of Dynamic Droplet Generation Processes

Xiurui Zhu^1^, Shisheng Su^1^, Mingzhu Fu^1^, Junyuan Liu^1^, Lingxiang Zhu^2,3^, Wenjun Yang^1,3^, Gaoshan Jing^4,*^, Yong Guo^1,*^

*Author affiliations:*

1. Department of Biomedical Engineering, School of Medicine, Collaborative Innovation Center for Diagnosis and Treatment of Infectious Diseases, Tsinghua University, Beijing, China.
2. National Research Institute for Family Planning, Beijing, China.
3. TargetingOne Corporation, Beijing, China.
4. Department of Precision Instrument, School of Mechanical Engineering, State Key Laboratory of Precision Measurement Technology and Instruments, Tsinghua University, Beijing, China.

** Corresponding authors:*

*Correspondence and requests for materials should be addressed to
Dr. Gaoshan Jing
Email: [gaoshanjing@mail.tsinghua.edu.cn](mailto:gaoshanjing@mail.tsinghua.edu.cn)
or
Dr. Yong Guo
Email: [yongguo@tsinghua.edu.cn](mailto:yongguo@tsinghua.edu.cn)

# Contents

[Supplementary tables 1](#_Toc516408174)

[**Table S1.** Data for droplet generation process in a stable condition in a single microfluidic channel. 1](#_Toc516408176)

[**Table S2.** Data for droplet generation process in a stable condition in multiple microfluidic channels. 3](#_Toc516408178)

[**Table S3.** Data for droplet generation process with artificial disturbances in a single microfluidic channel. 4](#_Toc516408180)

[**Table S4.** Data for pre-microgel droplet generation processes with or without artificial disturbances. 5](#_Toc516408182)

[**Table S5.** The pre-microgel droplet diameter’s mean values and CVs in the microscopic images. 6](#_Toc516408183)

[Supplementary methods 7](#_Toc516408184)

[A. The cosine similarity between two grayscale frames 7](#_Toc516408185)

[B. The Cosine Similarity Algorithm (CSA) method 8](#_Toc516408186)

[Appendix 13](#_Toc516408187)

[A. Mathematical proof 13](#_Toc516408188)

[B. User guide to software 19](#_Toc516408193)

[1. Droplet Frequency Meter software 19](#_Toc516408194)

[2. Droplet Property Analyzer software 25](#_Toc516408195)

[References 38](#_Toc516408196)

# Supplementary tables

### Case 1. Droplet generation process in a stable condition in a single microfluidic channel

Table S1. The flow rates, droplet generation frequencies, relative calculation errors and computational time of droplet generation processes in a stable condition in a single microfluidic channel. (CV: coefficient of variation, CT: computational time, SD: standard deviation)

| **Flow rate label** | **Oil flow rate (μL/h)** | **Water flow rate (μL/h)** | **Measured droplet generation frequency’s mean value (Hz)** | **Calculated droplet generation frequency’s mean value (Hz)** | **Relative calculation error** | **Droplet generation frequency’s CV** | **CT (s)** | **CT’s SD (s)** |
| --- | --- | --- | --- | --- | --- | --- | --- | --- |
| L1 | 1,000 | 500 | 90.1 | 90.0 | -0.08% | 1.37% | 1.63 | 0.02 |
| L2 | 1,200 | 600 | 111.4 | 111.5 | 0.05% | 0.91% | 1.63 | 0.02 |
| L3 | 1,500 | 750 | 147.6 | 147.6 | 0.01% | 0.73% | 1.63 | 0.01 |
| L4 | 2,000 | 1,000 | 211.7 | 211.7 | 0.00%^*^ | 0.37% | 1.64 | 0.02 |
| L5 | 2,500 | 1,250 | 278.0 | 278.0 | -0.03% | 0.66% | 1.64 | 0.02 |
| L6 | 3,000 | 1,500 | 389.7 | 389.7 | 0.00%^**^ | 1.48% | 1.63 | 0.01 |
| L7 | 3,500 | 1,750 | 477.4 | 477.0 | -0.07% | 0.97% | 1.62 | 0.01 |
| L8 | 4,000 | 2,000 | 562.5 | 562.8 | 0.04% | 0.75% | 1.63 | 0.02 |
| L9 | 4,500 | 2,250 | 685.1 | 685.6 | 0.06% | 1.34% | 1.63 | 0.01 |
| L10 | 5,000 | 2,500 | 776.1 | 776.0 | -0.01% | 1.89% | 1.64 | 0.03 |
| L11 | 5,500 | 2,750 | 941.4 | 941.3 | -0.01% | 1.62% | 1.62 | 0.02 |
| L12 | 6,000 | 3,000 | 1,053.4 | 1,053.6 | 0.02% | 0.73% | 1.64 | 0.02 |
| L13 | 6,500 | 3,250 | 1,126.1 | 1,125.8 | -0.02% | 0.77% | 1.64 | 0.01 |
| L14 | 7,000 | 3,500 | 1,326.4 | 1,325.9 | -0.03% | 0.94% | 1.63 | 0.01 |
| L15 | 7,500 | 3,750 | 1,481.6 | 1,481.9 | 0.03% | 0.37% | 1.64 | 0.00^†^ |
| L16 | 8,000 | 4,000 | 1,874.8 | 1,875.2 | 0.02% | 0.32% | 1.63 | 0.01 |
| L17 | 8,500 | 4,250 | 2,098.7 | 2,099.8 | 0.05% | 0.68% | 1.63 | 0.01 |
| L18 | 9,000 | 4,500 | 2,367.0 | 2,367.1 | 0.00%^***^ | 0.31% | 1.64 | 0.02 |
| L19 | 9,500 | 4,750 | 2,698.1 | 2,697.7 | -0.01% | 0.24% | 1.63 | 0.01 |
| L20 | 10,000 | 5,000 | 2,989.8 | 2,991.0 | 0.04% | 0.35% | 1.62 | 0.01 |
| L21 | 11,000 | 5,500 | 3,287.3 | 3,288.0 | 0.02% | 0.11% | 1.63 | 0.01 |
| L22 | 12,000 | 6,000 | 3,614.2 | 3,614.1 | 0.00%^****^ | 0.41% | 1.63 | 0.02 |
| L23 | 13,000 | 6,500 | 4,110.4 | 4,109.2 | -0.03% | 0.21% | 1.64 | 0.02 |
| L24 | 14,000 | 7,000 | 3,879.9 | 3,878.3 | -0.04% | 0.30% | 1.63 | 0.01 |
| L25 | 15,000 | 7,500 | 4,707.9 | 4,707.6 | -0.01% | 0.38% | 1.63 | 0.01 |
| R1 | 600 | 600 | 67.3 | 67.3 | -0.03% | 1.59% | 1.63 | 0.02 |
| R2 | 720 | 600 | 76.1 | 76.0 | -0.06% | 2.01% | 1.62 | 0.01 |
| R3 | 750 | 600 | 77.3 | 77.3 | -0.03% | 3.23% | 1.63 | 0.02 |
| R4 | 800 | 600 | 81.5 | 81.4 | -0.05% | 1.94% | 1.64 | 0.02 |
| R5 | 900 | 600 | 96.0 | 96.0 | -0.02% | 1.26% | 1.63 | 0.01 |
| R6 | 1,000 | 600 | 110.2 | 110.2 | 0.01% | 1.50% | 1.62 | 0.02 |
| R8 | 12,000 | 1,200 | 892.0 | 891.6 | -0.05% | 0.87% | 1.64 | 0.01 |
| R9 | 12,000 | 2,000 | 1,353.3 | 1,352.9 | -0.03% | 0.37% | 1.62 | 0.01 |
| R10 | 12,000 | 2,400 | 1,544.7 | 1,545.1 | 0.02% | 0.30% | 1.62 | 0.01 |
| R11 | 12,000 | 3,000 | 2,155.8 | 2,156.1 | 0.01% | 0.20% | 1.63 | 0.02 |
| R12 | 12,000 | 4,000 | 2,900.4 | 2,900.6 | 0.01% | 0.29% | 1.63 | 0.01 |
| R13 | 12,000 | 4,800 | 3,564.1 | 3,563.1 | -0.03% | 0.20% | 1.64 | 0.02 |

^*^ This value is -0.002% when more decimal places are shown.

^**^ This value is -0.003% when more decimal places are shown.

^***^ This value is 0.001% when more decimal places are shown.

^****^ This value is -0.001% when more decimal places are shown.

^†^ This value is 0.002 when more decimal places are shown.

### Case 2. Droplet generation process in a stable condition in multiple microfluidic channels

Table S2. The flow rates, droplet generation frequencies, relative calculation errors and computational time of droplet generation processes in a stable condition in multiple microfluidic channels and the corresponding values in a stable condition in a single microfluidic channel. (CT: computational time, SD: standard deviation)

| Video clip name | Oil flow rate (μL/h) | Water flow rate (μL/h) | Measured mean droplet generation frequency (Hz) | Calculated mean droplet generation frequency (Hz) | Relative calculation error | CT (s) | CT’s SD (s) |
| --- | --- | --- | --- | --- | --- | --- | --- |
| Low | 3,000 | 1,500 | 409.6 | 410.4 | 0.20% | 0.36 | 0.01 |
| Medium | 8,000 | 4,000 | 1,587.3 | 1,584.3 | -0.19% | 0.37 | 0.01 |
| High | 10,000 | 5,000 | 1,923.9 | 1,921.0 | -0.15% | 0.37 | 0.01 |
| Double channel  = Low  + Medium | 3,000 | 1,500 | 409.6 | 410.1 | 0.13% | 1.60 | 0.03 |
|  | 8,000 | 4,000 | 1,587.3 | 1,578.5 | -0.55% |  |  |
| Triple channel  = Low  + Medium  + High | 3,000 | 1,500 | 409.6 | 409.7 | 0.04% | 3.11 | 0.01 |
|  | 8,000 | 4,000 | 1,587.3 | 1,584.9 | -0.15% |  |  |
|  | 10,000 | 5,000 | 1,923.9 | 1,920.4 | -0.18% |  |  |

### Case 3. Droplet generation process with artificial disturbances in a single microfluidic channel

Table S3. The flow rates, droplet generation frequencies, relative calculation errors and computational time at different time points of a droplet generation process with artificial disturbances in a single microfluidic channel. The periods affected by the disturbances were determined by droplet generation frequency’s CV, and the CVs indicating the periods affected by the disturbances are highlighted in bold. (CV: coefficient of variation, CT: computational time, SD: standard deviation, ↑: flow rate increasing, ↓: flow rate decreasing)

| **Time (s)** | **Oil flow rate (μL/h)** | **Water flow rate (μL/h)** | **Measured droplet generation frequency’s mean value (Hz)** | **Calculated droplet generation frequency’s mean value (Hz)** | **Droplet generation frequency’s CV** | **CT (s)** | **CT’s SD (s)** |
| --- | --- | --- | --- | --- | --- | --- | --- |
| 301.25 | 2,000 | 1,000 | 188.5 | 188.4 | 1.04% | 1.65 | 0.03 |
| 331.25 | ↑ | 1,000 | 205.9 | 205.3 | **7.86%** | 1.63 | 0.02 |
| 333.75 | ↑ | 1,000 | 264.0 | 264.0 | **10.40%** | 1.64 | 0.02 |
| 338.75 | 4,000 | 1,000 | 310.7 | 310.8 | 1.24% | 1.64 | 0.03 |
| 346.25 | 4,000 | 1,000 | 318.0 | 318.0 | 1.52% | 1.64 | 0.02 |
| 361.25 | 4,000 | 1,000 | 297.1 | 297.0 | 1.74% | 1.64 | 0.01 |
| 391.25 | 4,000 | 1,000 | 309.9 | 309.9 | 3.01% | 1.64 | 0.02 |
| 421.25 | 4,000 | 1,000 | 312.6 | 312.5 | 1.22% | 1.65 | 0.02 |
| 631.25 | 4,000 | 1,000 | 314.5 | 314.5 | 1.06% | 1.63 | 0.02 |
| 661.25 | ↓ | 1,000 | 261.3 | 260.7 | **13.60%** | 1.64 | 0.02 |
| 663.75 | ↓ | 1,000 | 197.6 | 196.2 | **8.05%** | 1.65 | 0.03 |
| 668.75 | 1,000 | 1,000 | 148.8 | 148.6 | 3.49% | 1.64 | 0.02 |
| 676.25 | 1,000 | 1,000 | 127.7 | 127.7 | 3.24% | 1.64 | 0.01 |
| 691.25 | 1,000 | 1,000 | 117.9 | 118.0 | 0.91% | 1.63 | 0.02 |
| 721.25 | 1,000 | 1,000 | 109.5 | 109.5 | 0.79% | 1.64 | 0.02 |
| 751.25 | 1,000 | 1,000 | 111.2 | 111.2 | 0.35% | 1.64 | 0.01 |

### Case 4. Pre-microgel droplet generation processes with or without artificial disturbances

Table S4. The flow rates, droplet generation frequency’s mean values, their CVs and droplet diameter’s CVs at different time points of a pre-microgel droplet generation process with or without artificial disturbances. The period(s) affected by the disturbances were determined by droplet generation frequency’s CV and droplet diameter’s CV respectively, and the CVs indicating the period(s) affected by the disturbances are highlighted in bold.

| **A pre-microgel droplet generation process without disturbances** | | | | | |
| --- | --- | --- | --- | --- | --- |
| **Time (s)** | **Oil flow rate (μL/h)** | **Water flow rate (μL/h)** | **Droplet generation frequency’s mean value (Hz)** | **Droplet generation frequency’s CV** | **Droplet diameter’s CV** |
| 31.25 | 2,000 | 1,000 | 344.4 | 1.34% | 0.35% |
| 46.25 | 2,000 | 1,000 | 336.5 | 2.96% | 1.41% |
| 61.25 | 2,000 | 1,000 | 278.8 | 3.47% | 0.65% |
| 76.25 | 2,000 | 1,000 | 304.8 | 1.01% | 0.34% |
| 91.25 | 2,000 | 1,000 | 318.8 | 0.56% | 0.40% |
| **A pre-microgel droplet generation process with artificial disturbances** | | | | | |
| **Time (s)** | **Oil flow rate (μL/h)** | **Water flow rate (μL/h)** | **Droplet generation frequency’s mean value (Hz)** | **Droplet generation frequency’s CV** | **Droplet diameter’s CV** |
| 31.25 | 2,000 | 1,000 | 337.9 | 6.50% | 0.62% |
| 38.75 | 2,000 | 1,000 | 298.9 | 10.90% | 1.14% |
| 41.25 | 2,000 | 0 | 211.0 | **21.70%** | 2.45% |
| 43.75 | 2,000 | 0 | 113.4 | **18.70%** | 1.35% |
| 46.25 | 2,000 | 1,000 | 171.3 | **16.80%** | 2.49% |
| 51.25 | 2,000 | 1,000 | 311.9 | 1.23% | 0.55% |
| 58.75 | 2,000 | 1,000 | 283.6 | **22.00%** | **7.22%** |
| 61.25 | 0 | 1,000 | 181.0 | **20.70%** | **10.80%** |
| 63.75 | 2,000 | 1,000 | 274.6 | 9.57% | 3.13% |
| 76.25 | 2,000 | 1,000 | 361.6 | 0.95% | 0.45% |
| 91.25 | 2,000 | 1,000 | 316.4 | 1.89% | 0.53% |

Table S5. The pre-microgel droplet diameter’s mean values and CVs in the microscopic images.

| Image index | The pre-microgel droplet generation process without disturbances | | The pre-microgel droplet generation process with artificial disturbances | |
| --- | --- | --- | --- | --- |
|  | **Microgel diameter’s mean value (μm)** | **Microgel diameter’s CV** | **Microgel diameter’s mean value (μm)** | **Microgel diameter’s CV** |
| 1 | 115.0 | 2.62% | 119.9 | 7.17% |
| 2 | 116.5 | 2.44% | 117.0 | 6.42% |
| 3 | 116.4 | 2.57% | 118.5 | 7.46% |
| 4 | 116.9 | 3.11% | 116.3 | 5.05% |
| 5 | 115.4 | 3.06% | 116.6 | 5.03% |
| 6 | 115.8 | 2.61% | 116.2 | 9.07% |
| 7 | 116.7 | 2.98% | 116.0 | 5.95% |
| Mean | 116.1 | 2.77% | 117.2 | 6.59% |

# Supplementary methods

## Mathematical description of the CSA method

## A. The cosine similarity between two grayscale frames

Mathematically, the cosine similarity between two non-zero vectors $\mathbf{a}$ and $\mathbf{b}$ is defined as the cosine of the included angle $\left\langle\mathbf{a},\mathbf{b} \right\rangle$ ^1^, which is given by equation (1).

|  | $S\left( \mathbf{a},\mathbf{b} \right)\triangleq cos\left\langle\mathbf{a},\mathbf{b} \right\rangle=\frac{\mathbf{a}^{T}\mathbf{b}}{\left\Vert\mathbf{a} \right\Vert\left\Vert\mathbf{b} \right\Vert}$ | (1) |
| --- | --- | --- |

The range of cosine similarity is determined by the cosine function: $S\left( \mathbf{a},\mathbf{b} \right)\in\left[ -1,1 \right]$. The larger the $\left| S\left( \mathbf{a},\mathbf{b} \right) \right|$, the higher the non-zero vectors $\mathbf{a}$ and $\mathbf{b}$ are linearly correlated (“similar”).

The grayscales of the pixels in each frame can be put into a grayscale vector in a specific order. For example, for a grayscale frame (spatial resolution: $m\times n$ pixels) with index $i$ in which the grayscale of pixels are defined as $H\left( p,q,i \right), p=1,2,\cdots,n, q=1,2,\cdots,m$, the grayscale vector $\mathbf{h}\left( i \right)$ can be defined as equation (2).

| $\mathbf{h}\left( i \right)\boldsymbol{\triangleq}\left[ \begin{matrix} H\left( 1,1,i \right) \\ H\left( 1,2,i \right) \\ \vdots\\ H\left( 1,m,i \right) \\ H\left( 2,1,i \right) \\ H\left( 2,2,i \right) \\ \vdots\\ H\left( 2,m,i \right) \\ \vdots\\ H\left( p,q,i \right) \\ \vdots\\ H\left( n,1,i \right) \\ H\left( n,2,i \right) \\ \vdots\\ H\left( n,m,i \right) \end{matrix} \right]$ | (2) |
| --- | --- |

The cosine similarity between two grayscale frames is defined as the cosine similarity between their corresponding grayscale vectors. If one of the frames (with index $r$) is designated as the reference frame, the cosine similarity between each frame and the reference frame is given by the following equation.

| $S\left( i,r \right)\triangleq cos\left\langle\mathbf{h}\left( i \right),\mathbf{h}\left( r \right) \right\rangle=\frac{{\mathbf{h}\left( i \right)}^{T}\mathbf{h}\left( r \right)}{\left\Vert\mathbf{h}\left( i \right) \right\Vert\left\Vert\mathbf{h}\left( r \right) \right\Vert},i=1,2,\cdots,N_{\mathrm{Frames}}$ | (3) |
| --- | --- |

The grayscale ranges from 0 to 255 as integers. According to the range of cosine function, $S\left( i,r \right)\in\left[ 0, 1 \right].$ The higher the $S\left( i,r \right)$, the higher the non-zero vectors $\mathbf{h}\left( i \right)$ and $\mathbf{h}\left( r \right)$ are positively linearly correlated, the more similar the grayscale distributions are in the two frames, and the higher the similarity between the two frames. When $\mathbf{h}\left( i \right)$ and $\mathbf{h}\left( r \right)$ are positively linearly correlated, $S\left( i,r \right)$ reaches its maximum possible value 1.

It should be noted that cosine similarity is undefined when either of the grayscale vectors $\mathbf{h}\left( \cdot\right)=\mathbf{0}$, which corresponds to a monochromatic black frame. In this way, when calculating cosine similarity between frames, the grayscale vectors should be checked beforehand and zero grayscale vectors should always be avoided.

## B. The Cosine Similarity Algorithm (CSA) method

1. **Acquisition of droplet generation video clip at a constant frame rate**

The acquisition of droplet generation video clip is a temporal sampling of a droplet generation process. According to Nyquist-Shannon sampling theorem ^2^, the acquisition frame rate should be larger than two times the droplet generation frequency.

To determine appropriate acquisition frame rate $f_{\mathrm{Acq}}$, a rough estimation of droplet generation frequency is required. Briefly, acquire a droplet generation video clip for a short period of time (1–2 s) at the highest acquisition frame rate of the high-speed camera $f_{Acq,M\mathrm{ax}}$. Record the frame index $N_{Frames,1st}$ and $N_{Frames,2nd}$ in which the first and second droplets are generated respectively. Then the estimated droplet generation frequency $\hat{f}$ is given by equation (4).

|  | $\hat{f}=\frac{f_{Acq,M\mathrm{ax}}}{N_{Frames,2\mathrm{nd}}-N_{Frames,1\mathrm{st}}}$ | (4) |
| --- | --- | --- |

Droplet generation frequency increases with an increase in either the oil or water flow rate ^3^, and therefore the upper bound of the droplet generation frequency can be estimated with the frequency of a droplet generation process of higher oil and water flow rate. With the estimated droplet generation frequency $\hat{f}$, any acquisition frame rate $f_{\mathrm{Acq}}>2\hat{f}$ (preferably $f_{\mathrm{Acq}}>4\hat{f}$) can be used for the acquisition of video clip.

1. **Calculation of similarity vector from the droplet generation video clip**

One of the frames in the video clip is designated as the reference frame and given the index $r$. The cosine similarity between each frame of the video clip and the reference frame$S\left( i,r \right), i=1,2,\cdots,N_{\mathrm{Frames}}$ ($N_{\mathrm{Frames}}$ is the number of frames) can be calculated with equation (3). A similarity vector $\mathbf{s}$ can be constructed by putting the cosine similarities in ascending order of the frame index, as shown in equation (5). The similarity vector, as a waveform signal of frame index in vector form (Figure 1c), is used to characterize the change in the similarity between frames in the video clip and the reference frame along the timeline.

|  | $\mathbf{s}\triangleq\left[ \begin{aligned} s\left( 1 \right) \\ s\left( 2 \right) \\ \vdots\\ s\left( N_{\mathrm{Frames}} \right) \end{aligned} \right],s\left( i \right)\triangleq S\left( i,r \right), i=1,2,\cdots,N_{\mathrm{Frames}}$ | (5) |
| --- | --- | --- |

Droplet generation process in a stable condition is highly periodic. The reference frame will reappear periodically along the timeline in the video clip of a droplet generation process in a stable condition (Figure 1b). Every time the reference frame reappears, the corresponding cosine similarity reaches its local maximum, resulting in a peak in the similarity vector waveform (Figure 1c). Therefore, every peak in the similarity vector corresponds to the generation of a droplet. Droplet generation frequency equals the oscillating frequency of the corresponding similarity vector.

1. **Calculation of the similarity vector’s cyclic auto-spectrum**

Droplet generation frequency can be better revealed by the cyclic auto-spectrum of the similarity vector given in equation (6), calculated with fast Fourier transform (FFT).

|  | $p\left( f_{k} \right)\triangleq\left\vert\mathrm{FFT}\left[ \mathbf{s} \right] \right\vert^{2},f_{k}=\frac{kf_{\mathrm{Acq}}}{N_{\mathrm{Frames}}}\in\left( -\frac{f_{\mathrm{Acq}}}{2},\frac{f_{\mathrm{Acq}}}{2} \right),k\mathbb{\in Z}$ |  | (6) |
| --- | --- | --- | --- |

1. **Calculation of droplet generation frequency’s mean value and CV from the cyclic auto-spectrum**

With spectral analysis, droplet generation frequency’s mean value and CV can be calculated, describing droplet generation frequency in a comprehensive way. The spectral analysis consists of three steps: the calculation of fundamental frequency, the calculation of droplet generation frequency’s mean value and the calculation of droplet generation frequency’s CV.

1. **Droplet generation process in a single microfluidic channel**

In the cyclic auto-spectrum (Figure 1d), the fundamental frequency is the oscillating frequency of the similarity vector. It can be mathematically proved that the power of fundamental frequency $f^{*}$ is higher than that of any harmonic frequency, in the case of reasonable approximation (Appendix A of the supplementary information). If the direct current (DC) component $f_{0}$ of the auto-spectrum given by equation (6) is set at 0, the estimated fundamental frequency $\hat{f^{*}}$ can be extracted by calling the highest peak intensity in the auto-spectrum.

The droplet generation frequency’s mean value can be regarded as the center of the fundamental frequency peak. If the power density distribution around the fundamental frequency is taken into consideration, the calculation of the center can achieve a high accuracy. To avoid the harmonic frequencies in the auto-spectrum, a frequency band $F_{\mathrm{Band}}$ around the estimated fundamental frequency $\hat{f^{*}}$ is defined as equation (7). The following frequency analysis is performed in the range defined by the frequency band.

|  | $F_{\mathrm{Band}}\triangleq\left( 0.5\hat{f^{*}},m\mathrm{in}\left[ 1.5\hat{f^{*}},\frac{f_{\mathrm{Acq}}}{2} \right] \right)$ | (7) |
| --- | --- | --- |

To avoid disturbance from possible noise in the frequency band, a noise reduction threshold is defined as equation (8).

|  | $T_{\mathrm{Noise}}\triangleq0.01p\left( \hat{f^{*}} \right)$ | (8) |
| --- | --- | --- |

The droplet generation frequency’s mean value is calculated as the power-density-weighed mean of the frequency in $F_{\mathrm{Band}}$ whose power density is not lower than $T_{\mathrm{Noise}}$.

|  | $\bar{f}\triangleq\frac{\sum_{k} \left[ f_{k}\cdot p\left( f_{k} \right) \right]}{\sum_{k} \left[ p\left( f_{k} \right) \right]},f_{k}\in F_{\mathrm{Band}},p\left( f_{k} \right)\geq T_{\mathrm{Noise}}$ | (9) |
| --- | --- | --- |

The droplet generation frequency’s CV is calculated as the ratio of power-density-weighed SD of the fundamental frequency peak to the fundamental frequency center $\bar{f}$ in terms of the frequency in $F_{\mathrm{Band}}$ whose power density is not lower than $T_{\mathrm{Noise}}$.

|  | ${CV}_{f}\triangleq\frac{1}{\bar{f}}\sqrt{\frac{\sum_{k} \left[ \left( f_{k}-\bar{f} \right)^{2}\cdot p\left( f_{k} \right) \right]}{\sum_{k} \left[ p\left( f_{k} \right) \right]}},f_{k}\in F_{\mathrm{Band}},p\left( f_{k} \right)\geq T_{\mathrm{Noise}}$ | (10) |
| --- | --- | --- |

1. **Droplet generation process in multiple microfluidic channels**

Droplet generation process in multiple microfluidic channels may have multiple ($N_{\mathrm{Freq}}>1$) non-overlapping droplet generation frequencies. Every non-overlapping droplet generation frequency corresponds to a fundamental frequency $\hat{f^{*}}\left( i \right),i=1,2,\cdots,N_{\mathrm{Freq}}$ (in ascending order of fundamental frequency) in the auto-spectrum.

For the convenience of deduction, we define $\hat{f^{*}}\left( 0 \right)\triangleq0, \hat{f^{*}}\left( N_{\mathrm{Freq}}+1 \right)\triangleq\frac{f_{\mathrm{Acq}}}{2}$. The CSA method can be applied to a droplet generation process in multiple microfluidic channels by simply replacing equations (7)–(10) with equations (11)–(14) for the redefinition of frequency band, noise reduction threshold, and the droplet generation frequency’s mean values and CVs.

|  | $F_{\mathrm{Band}}\left( i \right)\triangleq\left( 0.5\hat{f^{*}}\left( i \right),\min\left[ 1.5\hat{f^{*}}\left( i \right),\frac{f_{\mathrm{Acq}}}{2} \right] \right)\cap\left( \frac{\hat{f^{*}}\left( i \right)+\hat{f^{*}}\left( i-1 \right)}{2},\frac{\hat{f^{*}}\left( i \right)+\hat{f^{*}}\left( i+1 \right)}{2} \right)$ | (11) |
| --- | --- | --- |
|  | $T_{\mathrm{Noise}}\left( i \right)\triangleq0.01p\left( \hat{f^{*}}\left( i \right) \right)$ | (12) |
|  | $\bar{f}\left( i \right)\triangleq\frac{\sum_{k} \left[ f_{k}\cdot p\left( f_{k} \right) \right]}{\sum_{k} \left[ p\left( f_{k} \right) \right]},f_{k}\in F_{\mathrm{Band}}\left( i \right),p\left( f_{k} \right)\geq T_{\mathrm{Noise}}\left( i \right)$ | (13) |
|  | ${CV}_{f}\left( i \right)\triangleq\frac{1}{\bar{f}\left( i \right)}\sqrt{\frac{\sum_{k} \left[ \left( f_{k}-\bar{f}\left( i \right) \right)^{2}\cdot p\left( f_{k} \right) \right]}{\sum_{k} \left[ p\left( f_{k} \right) \right]}},f_{k}\in F_{\mathrm{Band}}\left( i \right),p\left( f_{k} \right)\geq T_{\mathrm{Noise}}\left( i \right)$ | (14) |

# Appendix

## A. Mathematical proof

### Statement: in the cyclic auto-spectrum of a similarity vector, the power of fundamental frequency is higher than that of any harmonic frequency, if the curvatures at the ends of droplet plugs are ignored.

### Similarity vector can be approximated to a periodic triangle wave with flat bottoms and/or flat tops

If the curvatures at the ends of droplet plugs are ignored, the similarity vector calculated in the section “Principle of the CSA method” (Figure S1a) can be approximated to a periodic triangle wave with flat bottoms. With all cases taken into consideration, if the scope of the video clip can contain a complete droplet, the similarity vector is usually a periodic triangle wave with flat bottoms only; and if the scope of the video clip cannot contain a complete droplet, the similarity vector is a periodic triangle wave with flat bottoms and flat tops (Figure S1b). In conclusion, the similarity vector can be approximated to a periodic triangle wave with flat bottoms and/or flat tops.

To derive a universal proof that applies to different acquisition frame rates, the similarity vector is approximated to $s\left( t \right)$, a continuous function of time $t$. In this way, any similarity vector calculated with the CSA method can be regarded as a temporal sampling and truncation of $s\left( t \right)$. When the similarity vector contains many oscillation cycles (for example, 100–500 cycles in this paper), the power leakage caused by non-integer-period truncation is negligible, and therefore any cyclic auto-spectrum of the similarity vector can be accordingly regarded as a frequency sampling and truncation of the Fourier transform of $s\left( t \right)$.


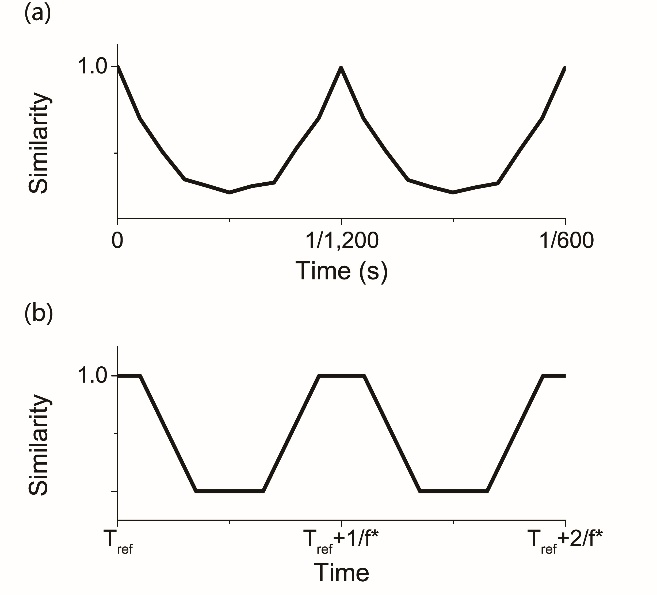


Figure S1. **An approximate mathematical description of the similarity vector.** The similarity vector of droplet generation video clips, including (a) the similarity vector as shown in Figure 1c, which can be approximated to (b) a periodic triangle wave with flat tops and/or flat bottoms by ignoring the curvatures at the ends of droplet plugs.

For a general mathematical description of the approximate similarity vector, consider a periodic triangle wave with period $T$ and amplitude $A$, whose tops are then flattened over $\varphi_{1}T$ and whose bottom are then fattened over $\varphi_{2}T$ in every period. If the reference frame corresponds to the time point $t_{\mathrm{Ref}}$ on the timeline of droplet generation video clip, the approximate similarity vector can be mathematically described as a periodic function consisting of four sections in each period (Figure S1b) shown in equation (15).

|  | $s\left( t \right)=\left\{ \begin{matrix} 1,t\in\left[ \left. t_{\mathrm{Ref}}+nT-\frac{\varphi_{1}T}{2},t_{\mathrm{Ref}}+nT+\frac{\varphi_{1}T}{2} \right) \right. \\ 1+2\varphi_{1}A-\frac{4A}{T}\left( t-t_{\mathrm{Ref}} \right),t\in\left[ \left. t_{\mathrm{Ref}}+nT+\frac{\varphi_{1}T}{2},t_{\mathrm{Ref}}+nT+\frac{\left( {1-\varphi}_{2} \right)T}{2} \right) \right. \\ 1-2\left( 1-\varphi_{1}-\varphi_{2} \right)A,t\in\left[ \left. t_{\mathrm{Ref}}+nT+\frac{\left( {1-\varphi}_{2} \right)T}{2},t_{\mathrm{Ref}}+nT+\frac{\left( {1+\varphi}_{2} \right)T}{2} \right) \right. \\ 1-2\left( {1-\varphi}_{1} \right)A+\frac{4A}{T}\left( t-\frac{T}{2}-t_{\mathrm{Ref}} \right),t\in\left[ \left. t_{\mathrm{Ref}}+nT+\frac{\left( {1+\varphi}_{2} \right)T}{2},t_{\mathrm{Ref}}+\left( n+1 \right)T-\frac{\varphi_{1}T}{2} \right) \right. \end{matrix} \right.,$  $T>0,A>0,\varphi_{1}\geq0,\varphi_{2}\geq0,\varphi_{1}+\varphi_{2}<1,n\mathbb{\in Z}$ | (15) |
| --- | --- | --- |

### On the cyclic auto-spectrum of a similarity vector, the power of fundamental frequency is higher than that of any harmonic frequency

Define $\delta\left( f \right)$ as unit pulse function centered at frequency $f$. In terms of the approximate similarity vector $s\left( t \right)$, if the fundamental frequency is defined as $f^{*}$ and the power of frequency component $kf^{*}$ is defined as $P\left( k \right)$, the auto-spectrum $p\left( f \right)$ of $s\left( t \right)$ is given by equation (16).

|  | $p\left( f \right)=P\left( 0 \right)\delta\left( 0 \right)+\sum_{k} P\left( k \right)\delta\left( kf^{*} \right)$ | (16) |
| --- | --- | --- |

If the Fourier coefficients of the periodic function $s\left( t \right)$ are $a_{k}$, then $P\left( k \right)$ is given by equation (17).

|  | $P\left( k \right)=\left\vert\frac{a_{k}}{f^{*}} \right\vert^{2},k\neq0,k\mathbb{\in Z}$ | (17) |
| --- | --- | --- |

The statement to be proved can be rewritten as equation (18).

|  | $\forall\left\vert k \right\vert\geq2,k\mathbb{\in Z};P\left( 1 \right)>P\left( k \right)$ | (18) |
| --- | --- | --- |

According to equation (17), the statement to be proved can be further rewritten as equation (19).

|  | $\forall\left\vert k \right\vert\geq2,k\mathbb{\in Z};\left\vert a_{1} \right\vert>\left\vert a_{k} \right\vert$ | (19) |
| --- | --- | --- |

The Fourier coefficients $a_{k},k\neq0,k\mathbb{\in Z}$ are given by equation (20).

|  | $a_{k}\triangleq\frac{1}{T}\int_{t_{\mathrm{Ref}}}^{t_{\mathrm{Ref}}+T} s\left( t \right)\exp\left( -jk\frac{2\pi}{T}t \right)dt$ $=\frac{2A}{k^{2}\pi^{2}}\left[ \cos\left( k\pi\varphi_{1} \right)-\left( -1 \right)^{k}\cos\left( k\pi\varphi_{2} \right) \right]$ | (20) |
| --- | --- | --- |

Considering the ranges of $\varphi_{1}+\varphi_{2}$ and $\left| \varphi_{1}-\varphi_{2} \right|$ according to the criteria $\varphi_{1}\geq0,\varphi_{2}\geq0,\varphi_{1}+\varphi_{2}<1$ in equation (15), we have equation (21).

|  | $\varphi_{1}+\varphi_{2}\in\left[ \left. 0,1 \right) \right.,\left\vert\varphi_{1}-\varphi_{2} \right\vert\in\left[ 0,\varphi_{1}+\varphi_{2} \right]\subseteq\left[ \left. 0,1 \right) \right.$ | (21) |
| --- | --- | --- |

Therefore, according to equation (22), the Fourier coefficient can be used as denominative.

|  | $a_{1}=\frac{2A}{\pi^{2}}\left[ \cos\left( \pi\varphi_{1} \right)+\cos\left( \pi\varphi_{2} \right) \right]$ $=\frac{4A}{\pi^{2}}\cos\left( \pi\frac{\varphi_{1}+\varphi_{2}}{2} \right)\cos\left( \pi\frac{\varphi_{1}-\varphi_{2}}{2} \right)\neq0$ | (22) |
| --- | --- | --- |

Therefore the inequality in equation (19) can be equally converted to equation (23).

|  | $\left\vert a_{1} \right\vert>\left\vert a_{k} \right\vert\Longleftrightarrow\frac{\left\vert a_{k} \right\vert}{\left\vert a_{1} \right\vert}<1$ | (23) |
| --- | --- | --- |

Given that $\left( -1 \right)^{k},k\mathbb{\in Z}$ in equation (20) alternates between 1 and -1 with different $k$, equation (19) can be equally converted to a set of three equations (24)–(26), which can be proved separately.

|  | $\frac{\left\vert a_{2} \right\vert}{\left\vert a_{1} \right\vert}<1$ | (24) |
| --- | --- | --- |
|  | $\forall\left\vert k \right\vert\geq4,k is even;\left\vert a_{k} \right\vert\leq\left\vert a_{2} \right\vert$ | (25) |
|  | $\forall\left\vert k \right\vert\geq3,k is odd;\frac{\left\vert a_{k} \right\vert}{\left\vert a_{1} \right\vert}<1$ | (26) |

- - 1. **Proof of equation (24)**

|  | $\frac{\left\vert a_{2} \right\vert}{\left\vert a_{1} \right\vert}=\left\vert\frac{\cos\left( 2\pi\varphi_{1} \right)-\cos\left( 2\pi\varphi_{2} \right)}{4\left[ \cos\left( \pi\varphi_{1} \right)+\cos\left( \pi\varphi_{2} \right) \right]} \right\vert$ $=\frac{1}{2}\left\vert\cos\left( \pi\varphi_{1} \right)-\cos\left( \pi\varphi_{2} \right) \right\vert$ $=\left\vert\sin\left( \pi\frac{\varphi_{1}+\varphi_{2}}{2} \right) \right\vert\left\vert\sin\left( \pi\frac{\varphi_{1}-\varphi_{2}}{2} \right) \right\vert\leq1$ | (27) |
| --- | --- | --- |

If and only if $\varphi_{1}$ and $\varphi_{2}$ meet the criterion set in equation (28) at the same time, the equality in equation (27) is established.

|  | $\left\{ \begin{matrix} \pi\frac{\varphi_{1}+\varphi_{2}}{2}=n_{1}\pi+\frac{\pi}{2},n_{1}\mathbb{\in Z} \\ \pi\frac{\varphi_{1}-\varphi_{2}}{2}=n_{2}\pi+\frac{\pi}{2},n_{2}\mathbb{\in Z} \\ \varphi_{1}\geq0,\varphi_{2}\geq0,\varphi_{1}+\varphi_{2}\in\left[ \left. 0,1 \right) \right. \end{matrix} \right.$ | (28) |
| --- | --- | --- |

There is no solution to the criterion set in equation (28), and therefore the equality in equation (27) cannot be established. Equation (24) is proved.

- - 1. **Proof of equation (25)**

Consider the case $a_{2}=0$, in which $a_{k}$ is given by equation (29).

|  | $\forall\left\vert k \right\vert\geq4,k is even;a_{k}=\frac{2A}{k^{2}\pi^{2}}\left[ \cos\left( k\pi\varphi_{1} \right)-\cos\left( k\pi\varphi_{1} \right) \right]=0$ | (29) |
| --- | --- | --- |

Therefore, this case conforms with the equality in equation (25).

Consider the case $a_{2}\neq0$, in which $\frac{\left| a_{k} \right|}{\left| a_{2} \right|}$ is given by equation (30).

|  | $\frac{\left\vert a_{k} \right\vert}{\left\vert a_{2} \right\vert}=\left\vert\frac{4\left[ \cos\left( k\pi\varphi_{1} \right)-\cos\left( k\pi\varphi_{2} \right) \right]}{k^{2}\left[ \cos\left( 2\pi\varphi_{1} \right)-\cos\left( 2\pi\varphi_{2} \right) \right]} \right\vert$ $=\left\vert\frac{\sin\left[ \frac{k\pi}{2} \left( \varphi_{1}+\varphi_{2} \right) \right]}{\frac{k}{2}\sin\left[ \pi\left( \varphi_{1}+\varphi_{2} \right) \right]} \right\vert\left\vert\frac{\sin\left[ \frac{k\pi}{2} \left( \varphi_{1}-\varphi_{2} \right) \right]}{\frac{k}{2}\sin\left[ \pi\left( \varphi_{1}-\varphi_{2} \right) \right]} \right\vert$ | (30) |
| --- | --- | --- |

The trigonometric inequality ^4^ in equation (31) can be used to prove $\frac{\left| a_{k} \right|}{\left| a_{2} \right|}\leq1$.

|  | $\forall x\mathbb{\in R,}m\mathbb{\in Z;}\left\vert\sin\left( mx \right) \right\vert\leq\left\vert m\sin x \right\vert$ | (31) |
| --- | --- | --- |

For $m\geq2$, the equality in (31) is established if and only if $x=n\pi,n\mathbb{\in Z}$.

Put $x=\frac{\pi}{2}\left( \varphi_{1}\pm\varphi_{2} \right),m=k$ into equation (31), which results in equation (32).

|  | $\forall\left\vert k \right\vert\geq4,k is even,a_{2}\neq0;\frac{\left\vert a_{k} \right\vert}{\left\vert a_{2} \right\vert}\leq1$ | (32) |
| --- | --- | --- |

According to equations (29) and (32), equation (25) is proved.

- - 1. **Proof of equation (26)**

|  | $\frac{\left\vert a_{k} \right\vert}{\left\vert a_{1} \right\vert}=\left\vert\frac{\cos\left( k\pi\varphi_{1} \right)+\cos\left( k\pi\varphi_{2} \right)}{k^{2}\left[ \cos\left( \pi\varphi_{1} \right)+\cos\left( \pi\varphi_{2} \right) \right]} \right\vert$ $=\left\vert\frac{\cos\left[ \frac{k\pi}{2} \left( \varphi_{1}+\varphi_{2} \right) \right]}{\frac{k}{2}\cos\left[ \frac{\pi}{2}\left( \varphi_{1}+\varphi_{2} \right) \right]} \right\vert\left\vert\frac{\cos\left[ \frac{k\pi}{2} \left( \varphi_{1}-\varphi_{2} \right) \right]}{\frac{k}{2}\cos\left[ \frac{\pi}{2}\left( \varphi_{1}-\varphi_{2} \right) \right]} \right\vert$ | (33) |
| --- | --- | --- |

Put $x=y+\frac{\pi}{2}$ into equation (31), we have equation (34).

|  | $\forall y\mathbb{\in R,}m is odd;\left\vert\cos\left( my \right) \right\vert\leq\left\vert m\cos y \right\vert$ | (34) |
| --- | --- | --- |

For $m\geq3$, the equality in equation (34) is established if and only if $y=n\pi+\frac{\pi}{2},n\mathbb{\in Z}$.

Put $y=\frac{\pi}{2}\left( \varphi_{1}\pm\varphi_{2} \right),m=k$ into equation (34), we have equation (35).

|  | $\forall\left\vert k \right\vert\geq3,k is odd;\frac{\left\vert a_{k} \right\vert}{\left\vert a_{1} \right\vert}\leq1$ | (35) |
| --- | --- | --- |

If and only if $\varphi_{1}$ and $\varphi_{2}$ meet the criterion set in equation (36) at the same time, the equality in equation (35) is established.

|  | $\left\{ \begin{matrix} \pi\frac{\varphi_{1}+\varphi_{2}}{2}=n_{1}\pi+\frac{\pi}{2},n_{1}\mathbb{\in Z} \\ \pi\frac{\varphi_{1}-\varphi_{2}}{2}=n_{2}\pi+\frac{\pi}{2},n_{2}\mathbb{\in Z} \\ \varphi_{1}\geq0,\varphi_{2}\geq0,\varphi_{1}+\varphi_{2}\in\left[ \left. 0,1 \right) \right. \end{matrix} \right.$ | (36) |
| --- | --- | --- |

There is no solution to the criterion set in equation (36), and therefore the equality in equation (35) cannot be established. Therefore, equation (26) is proved.

In summary of the equation set (24)–(26), equation (19) is proved, which is equivalent to the statement to be proved.

## B. User guide to software

### Droplet Frequency Meter software

The Droplet Frequency Meter software provides a method to calculate the distribution of droplet generation frequencies as well as their mean values and CVs, based on the CSA method introduced in this paper. The software can be further used to view and crop video clips (for rough estimation of droplet generation frequencies and selection of the desired region of interest) and export calculation results.

As a video clip used in this paper takes up quite a large amount of RAM, it is suggested that the Droplet Frequency Meter software should be run on a 64-bit operation system such as Microsoft Windows 7 Premium (64-bit), with at least 8 GB RAM (preferably 16 GB RAM). It is also advisable to close irrelevant software prior to a run.

In this software, the first step is to load a droplet generation video clip. Next, the video clip is cropped in terms of resolution and timeline if necessary. Then, the reference frame is designated. After that, the computational parameters are configured. Finally, the droplet generation frequency’s distribution is calculated, revealing its mean value and CV as computational results. Computational time of the CSA method can be evaluated in the software, and calculation results including data and plots can be exported for further analysis. These steps are further elaborated in the following sections.

**Step 1. Load a droplet generation video clip**

Open the software.


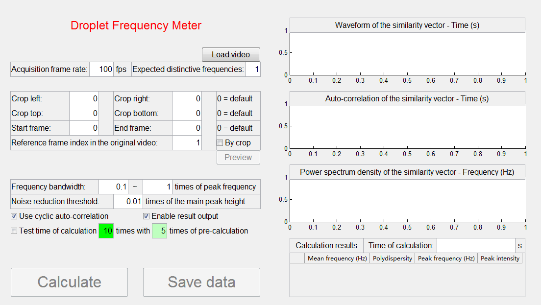


Click the “Load video” button, which opens a dialog to load a file.


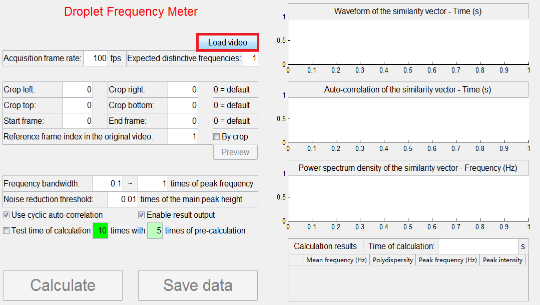


Select a droplet generation video clip (AVI files, preferably with uncompressed encoding or encoded with VideoWriter module in Matlab for the best compatibility) in the pop-up open dialog, and click the “Open” button. Then the video is loaded into the preview area.


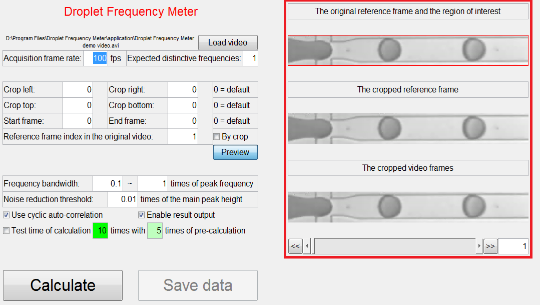


**Step 2. Crop the video clip in terms of resolution and timeline (not used in this paper)**

The video clip may be cropped in terms of resolution (width and height) and timeline if necessary, by setting the edges of the frames and the start and end frames of the video clip. The effect of cropping is shown as below, but this step was not performed in this paper.


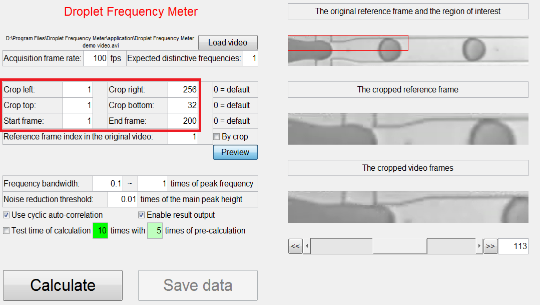


**Step 3. Designate the reference frame**

The index of the reference frame is designated in the text box shown in the red box below. If the “By crop” is toggled to the “on” state, the start frame of the cropped video clip is defined as frame 1, and otherwise the start frame of the original video clip is defined as frame 1, which means that the reference frame is NOT necessarily a frame in the cropped video clip.


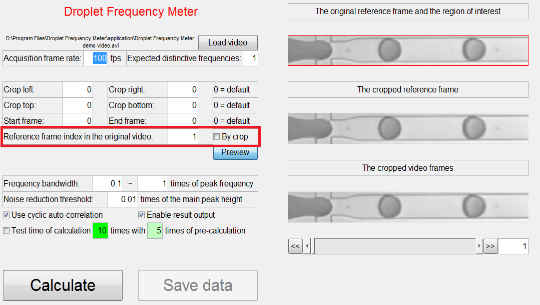


**Step 4. Set the computational parameters**

Computational parameters include acquisition frame rate, number of expected distinctive frequencies, frequency bandwidth, noise reduction, cyclic auto-correlation and auto-spectrum, plot enabling and test of computational time. Among these parameters, the frequency bandwidth, noise reduction, cyclic auto-correlation and auto-spectrum and plot enabling are often set as default. The rest should be configured according to the following suggestions.

The acquisition frame rate is set at the frame rate when the video clip is acquired, for example, the frame rate of the high-speed camera. It is NOT necessarily the frame rate when the video clip is played.

The number of expected distinctive frequencies is set at the number of non-overlapping droplet generation frequencies in the video clip. For a droplet generation process in a single microfluidic channel, this parameter is set at 1.


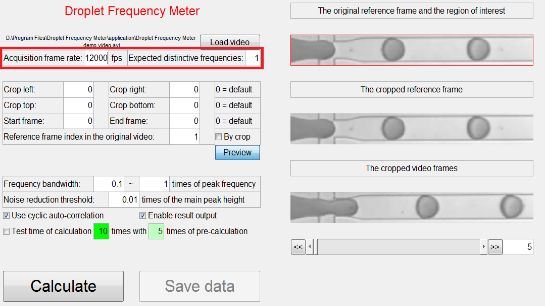


The time of calculation testing is used to evaluate the computational time of CSA. When toggled to the “on” state, the text boxes in the line set the number of repetition during the evaluation and the number of repetition before the evaluation. Repeating the calculation before the evaluation of computational time puts the run of the software in a stable condition when the computational time is evaluated.


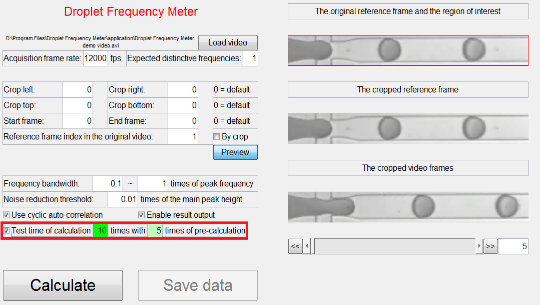


**Step 5. Calculate droplet generation frequency’s distribution, mean value, and CV**

Click the “Calculation” button.


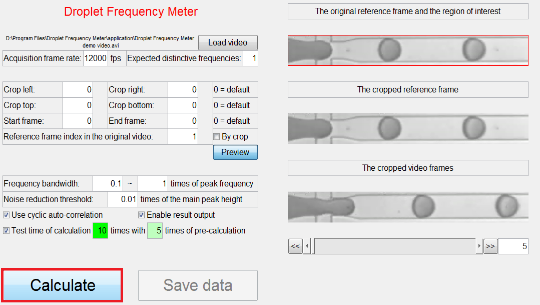


At the end of the calculation, the similarity vector, its auto-correlation and auto-spectrum is plotted in the result area, and the computational time and spectral analysis results are listed in the table below.


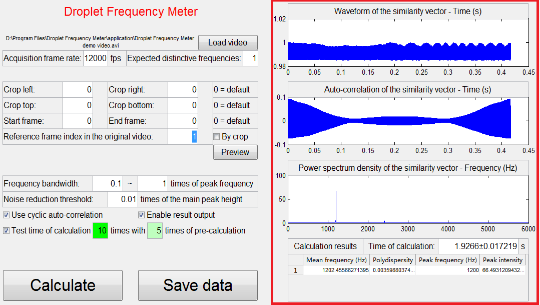


**Step 6. Export calculation results and plot data**

The calculation results can be exported by clicking the “Save data” button, which will open a pop-up save dialog and export the data to a text file (*.txt).


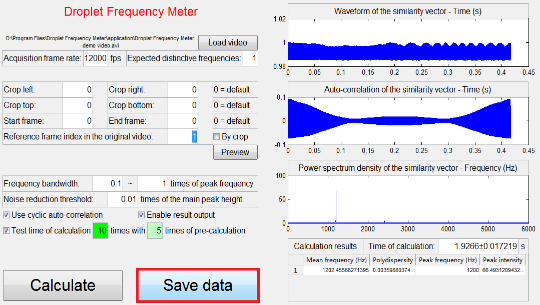


### Droplet Property Analyzer software

The Droplet Property Analyzer software provides a method to perform statistical analysis on droplet diameters.

In this software, the first step is to load a microscopic image of droplets. Next, the range of droplet diameter is estimated. Then, the scale of the pixel is determined. After that, the borders of the droplets are recognized. Finally, the statistical values (for example, the mean, SD, and CV) of the droplet diameters are automatically calculated and scaled to the designated scale. Viewing and deleting of any droplet border (and its corresponding diameter) are further enabled in case of droplets with abnormal sizes, but in this paper, this step was skipped because there were no such abnormal droplets in the microscopic images. Data processing methods such as exporting and importing images and data are provided as well. These steps are further elaborated in the following sections.

**Step 1. Load a microscopic image of droplets**

Open the software.


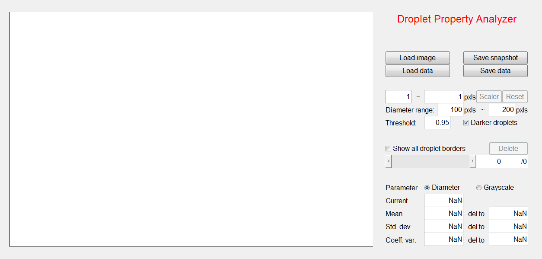


Click the “Load image” button, which opens a dialog to load a file.


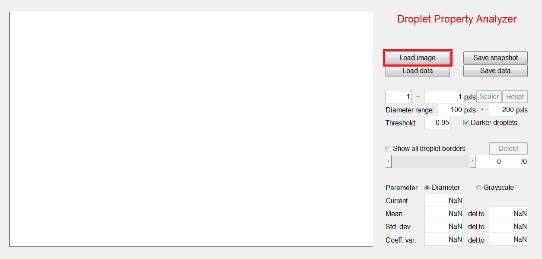


Select a microscopic image of droplets in the pop-up open dialog, and the “Open” button. Then the image is loaded into the image area of the software. Droplets may be recognized upon loading if the default parameters are proper for border recognition.


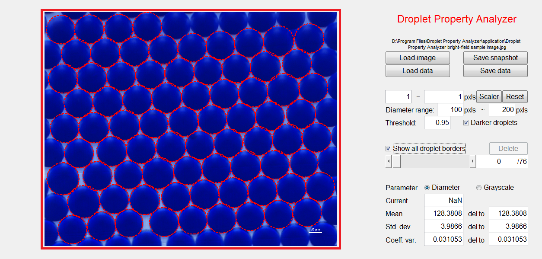


**Step 2. Estimate the range of droplet diameter**

Click the “Scaler” toggle button, which sets the button to the “on” state and enables the drawing of a line in the image area.


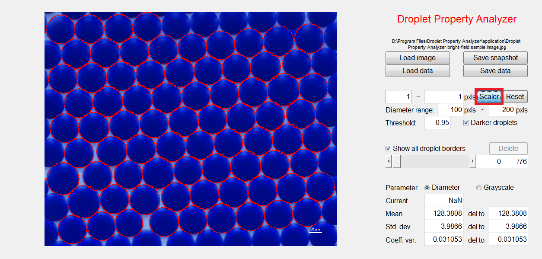


The start and end of the line may be adjusted until they fit well with the diameter of a droplet.


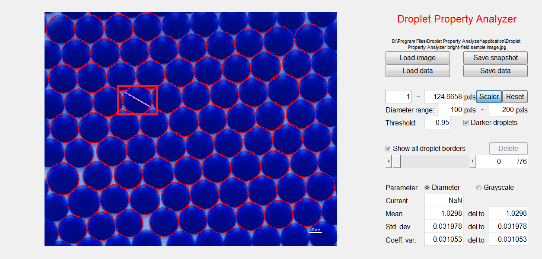


The estimated diameter in pixels is shown in the text box to the left of the “Scaler” button.


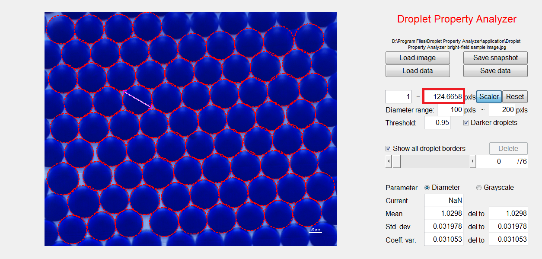


Click the “Scaler” toggle button again, which sets the button to the “off” state, clears the line drawn in the image area, and disables drawing in the image area.


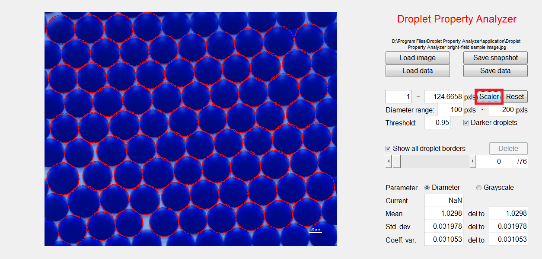


To achieve a fast calculation, it is suggested that the range of diameter not be more than 200 pixels. To achieve a high accuracy, it is suggested that the diameters of multiple droplets be measured.

**Step 3. Determine the scale in pixel**

Click the “Scaler” button again, which sets the button to the “on” state and enables the drawing of a line in the image area.


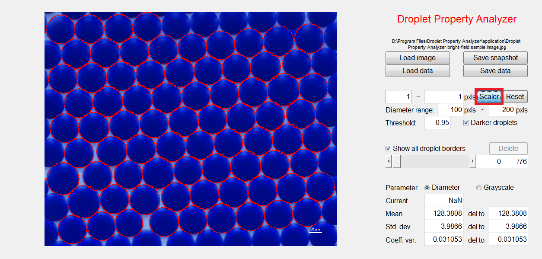


At this time, draw a line to any object in the image with known distance metric (for example, the scale bar in the image reading 50 μm or the length of a grid known to be 200 μm). The start and end of the line may be adjusted until they fit well with the designated object.


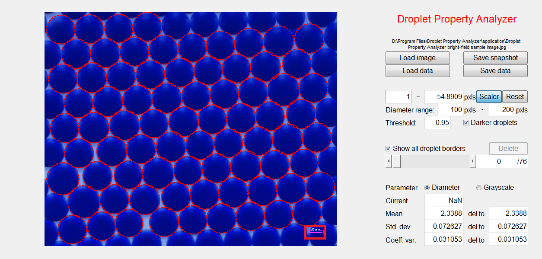


The distance in pixels is shown in the text box to the left of the “Scaler” button.


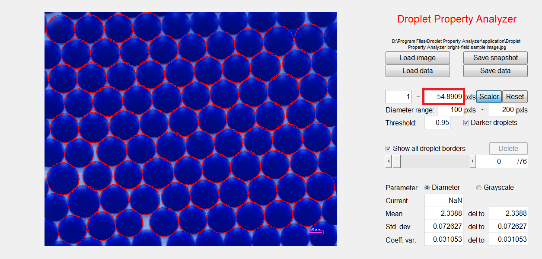


Key the distance metric in a known unit into the text box on the left side of the equal sign, and then press “Enter”. For example, the scale bar in the image is known as 50 μm, and thus key the distance metric 50 into the text box so that the results are converted to the known unit of μm after calculation.


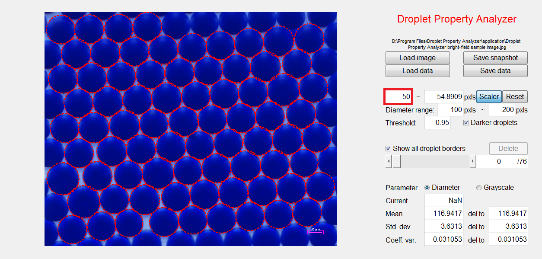


Click the “Scaler” toggle button again, which sets the button to the “off” state, clears the line drawn in the image area and disables drawing in the image area.


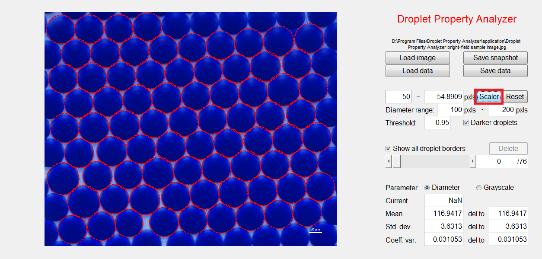


If another measurement of the scale is required, press the “reset” button (which resets both values in the text boxes to 1) and repeat the steps above in this section.


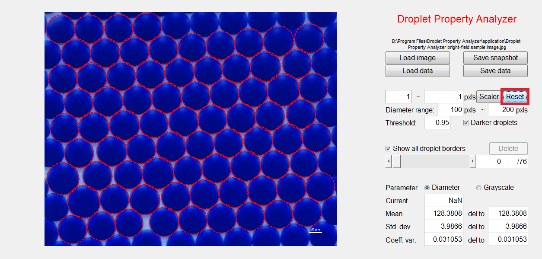


**Step 4. Recognize the circular borders of droplets**

With reference to the estimated droplet diameter, the range of diameters can be set at 100–150 pixels (press “Enter” key for each change to take effect). The threshold is suggested to be in the range of 0.94–0.96 for optimal recognition results, and one may start with the suggested value 0.95. As the droplets are darker than the background, the “Darker droplets” option is toggled to the “on” state. With all these settings, the borders of the droplets should be recognized by the software. To achieve a high accuracy, it is designed in the software that only droplets with complete circular borders are recognized.


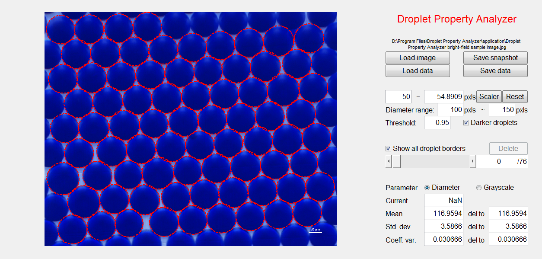


The statistical values of droplet diameters are listed in the bottom left column of text boxes.


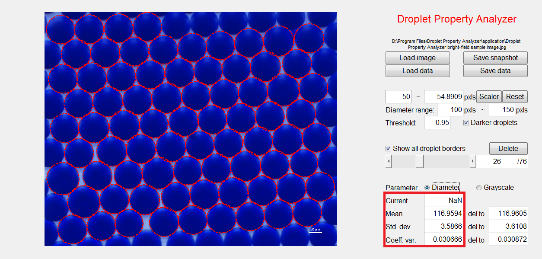


**Step 5. View and delete a droplet border and its corresponding diameter (not used in this paper)**

To view any droplet border, first, toggle the “Show all droplet borders” to the “off” state. Then, three options are provided to individually view the diameter of any droplet.

The first and most commonly used option is to simply click on the droplet in the image.


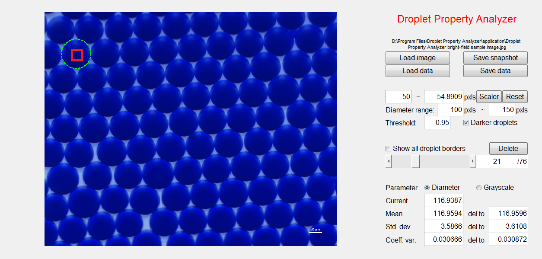


The second option is to scroll the bar in the middle of the column to the right.


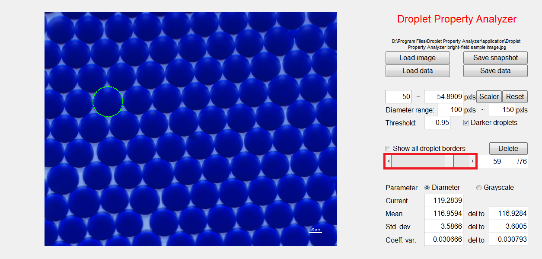


The third option is to directly key the index into the text box to the right of the scrollbar.


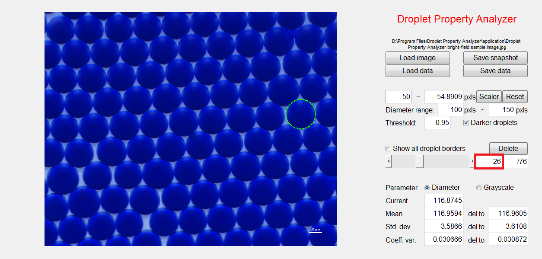


The diameter of the current droplet (in green circle) is displayed in the text box to the right of “Current” (for example, 116.9 μm for droplet #21, 119.3 μm for droplet #59 and 116.9 μm for droplet # 26).

To delete a droplet border, select a droplet with either of the three options above, and then simply click the “Delete” button.


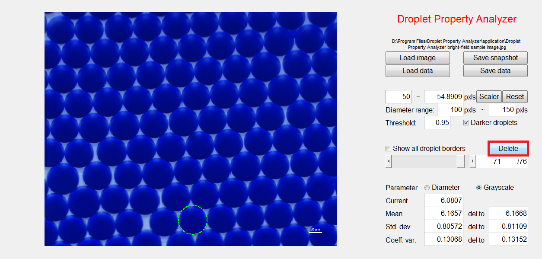


To preview the effect of deleting the currently selected droplet border, the statistic values with the currently selected droplet border deleted are listed in the bottom right column of text boxes.


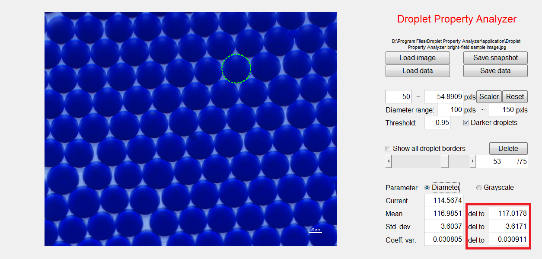


**Step 6. Snapshots, data exporting and importing**

The image with recognized droplet borders can be exported by clicking the “Save snapshot” button, which will open a dialog and export the data to a file in a designated format. Jpeg (*.jpg), bitmap (*.bmp) and tiff (*.tif) formats are provided as options.


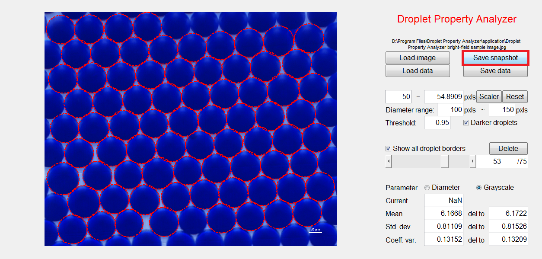


The diameters of the droplets and their statistic values can be exported by clicking the “Save data” button, which will export the data to a text (*.txt) file.


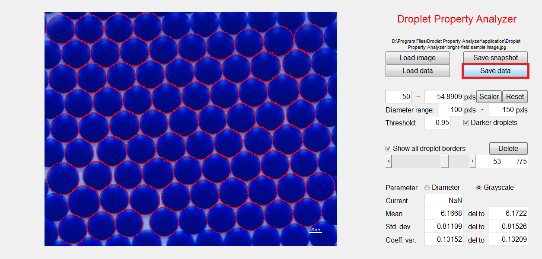


To continue the process with an exported data file, click the “Load data” button to import the data from a saved text (*.txt) file and continue processing.


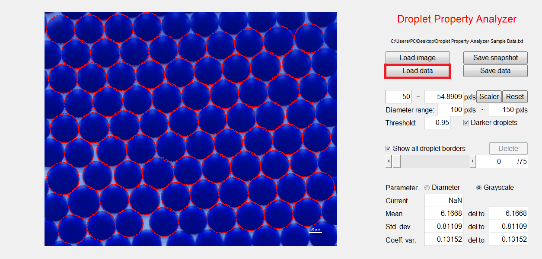


# References

1 Fred, A., de Marsico, M. & Figueiredo, M. Pattern recognition: applications and methods: 4^th^ international conference, ICPRAM, revised selected papers. 115 (Springer, 2016).

2 Marks, R. Introduction to Shannon sampling and interpolation theory. 2 (Springer Science & Business Media, 2012).

3 Yobas, L., Martens, S., Ong, W.-L. & Ranganathan, N. High-performance flow-focusing geometry for spontaneous generation of monodispersed droplets. *Lab Chip* **6**, 1073-1079 (2006).

4 Pedrick, G. A first course in analysis. 7 (Springer Science & Business Media, 2012).
